# ﻿Revision of *Acroclisoides* Girault & Dodd, 1915 (Chalcidoidea, Pteromalidae, Metasteninae)

**DOI:** 10.3897/zookeys.1257.157985

**Published:** 2025-10-30

**Authors:** Ekaterina V. Tselikh, Mircea-Dan Mitroiu, Jaehyeon Lee, Natalie Dale-Skey, Deok-Seo Ku

**Affiliations:** 1 Zoological Institute, Russian Academy of Sciences, St. Petersburg 199034, Russia Zoological Institute, Russian Academy of Sciences St. Petersburg Russia; 2 Faculty of Biology, Alexandru Ioan Cuza University, Iasi, Romania Alexandru Ioan Cuza University Iasi Romania; 3 Department of Plant Medicine, Gyeongsang National University, Jinju 52828, Republic of Korea Gyeongsang National University Jinju Republic of Korea; 4 Natural History Museum, London, UK Natural History Museum London United Kingdom; 5 The Science Museum of Natural Enemies, Geochang 50147, Republic of Korea The Science Museum of Natural Enemies Geochang Republic of Korea

**Keywords:** Description, key, Metasteninae, new record, new species, parasitoid, redescription

## Abstract

The species of *Acroclisoides* Girault & Dodd (Pteromalidae: Metasteninae) are revised. Twenty-three world species are recognized based on females, of which eight new species are described: *Acroclisoides bimaculatus* Tselikh, **sp. nov.** (Australia), *A. fusus* Tselikh & Mitroiu, **sp. nov.** (Africa), *A. marimbae* Tselikh & Mitroiu, **sp. nov.** (Africa), *A. miklukhai* Tselikh, **sp. nov.** (Papua New Guinea), *A. nongae* Tselikh, Lee & Ku, **sp. nov.** (Eastern Palearctic), *A. simbis* Tselikh & Mitroiu, **sp. nov.** (Africa), *A. supramaculatus* Tselikh, **sp. nov.** (Australia) and *A. suryai* Tselikh, **sp. nov.** (India). The genus *Golovissima* Dzhanokmen, 1982, **syn. nov.**, is synonymized under *Acroclisoides* Girault & Dodd, 1915; the species *Euneura borocerae* Risbec, 1957, previously included in *Acroclisoides* by Bouček (1976), is transferred to *Agiommatus* Crawford, 1911, as *A. borocerae* (Risbec, 1957), **comb. nov.** The hitherto unknown male of *A. major* Girault & Dodd, 1915 is described for the first time. The following new records are reported: *A. laticeps* Girault & Dodd, 1915 from Papua New Guinea; *A. luzonensis* Gahan, 1920 from Indonesia, Malaysia and Republic of Korea; *A. sinicus* (Huang & Liao, 1988) from Japan and Vietnam; *A. solus* Grissell & Smith, 2006 from Japan and Russia. All the species are described or redescribed and illustrated by macrophotography, including type material of previously described species, and a key for the identification of the twenty-three known species is given.

## ﻿Introduction

The pteromalid genus *Acroclisoides* (type species *Acroclisoides megacephalus* Girault & Dodd, 1915) belongs to the family Pteromalidae, subfamily Metasteninae (Burks et al. 2022). We are using Metasteninae Ashmead, 1904 instead of Pachyneurinae Ashmead, 1904, as the latter name is a junior homonym of Pachyneurinae Schiner, 1864 (Diptera). Up to now, it comprised 15 species worldwide (UCD Community 2025). Three of the known species, *Acroclisoides africanus* Ferrière, 1940, *A. borocerae* (Risbec, 1957) and *A. spilopterus* (Masi, 1917), inhabit the Afrotropical region. Four species, *Acroclisoides laticeps* Girault & Dodd, 1915, *A. major* Girault & Dodd, 1915, *A. megacephalus* Girault & Dodd, 1915 and *A. tectacorisi* (Girault, 1924), are distributed in the Australian region. Six other species, *Acroclisoides indicus* Ferrière, 1931, *A. luzonensis* Gahan, 1920, *A. maculatus* Sureshan & Narendran, 2002, *A. quintus* Xiao & Huang, 2000, *A. sativus* Kumar & Khan, 2012 and *A. sinicus* (Huang & Liao, 1988), are distributed in the Oriental region. Three species, *Acroclisoides bicolor* Luo & Qin, 1991, *A. emeljanovi* Dzhanokmen, 1982 and *A. sinicus* (Huang & Liao, 1988), are known from Palearctic region. *Euneura borocerae* Risbec, 1957 was transferred to *Acroclisoides* by Bouček (1976), but the examination of the type specimens in MNHN showed that the species in fact belongs to *Agiommatus* Crawford, 1911 as *Agiommatus borocerae* (Risbec, 1957), comb. nov.

The biology of most *Acroclisoides* species is known: they are egg parasitoids of hemipteran genera of the family Pentatomidae: *Agonoscelis* Spinola, 1837, *Antestiopsis* Leston, 1952, *Atelocera* Laporte, 1832, *Axiagastus* Dallas, 1851, *Bathycoelia* Amyot & Serville, 1843, *Biprorulus* Breddin, 1900, *Erthesina* Spinola, 1837, *Halyomorpha* Mayr, 1864, *Nezara* Amyot & Serville, 1843, *Oechalia* Stål, 1862, *Placosternum* Amyot & Serville, 1843, *Plautia* Stål, 1864 (Thompson 1958; Herting 1971; Thirumalai and Ananthakrishnan 1977; Luo and Qin 1991; Clarke and Seymour 1992; Coombs and Khan 1998; Field et al. 1998; Xiao and Huang 2000; Grissell and Smith 2006; UCD Community 2025) and of the family Acanthosomatidae: *Acanthosoma* Curtis, 1824 (Dzhanokmen 1982). Also, *Acroclisoides* includes species that are apparently facultative or obligate hyperparasitoids of scelionids of the genera *Aphanurus* Looss, 1907, *Asolcus* Miyake, 1900, *Telenomus* Haliday, 1833 and *Trissolcus* Ashmead, 1893 (Baltazar 1966; Grissell and Smith 2006; Sabbatini et al. 2019; UCD Community 2025) and also of eupelmids of the genus *Anastatus* Motschulsky, 1859 (Grissell and Smith 2006; Sabbatini et al. 2019; UCD Community 2025).

This paper revises and keys the known species of *Acroclisoides* based on examination of all available material including types, redescribes the previously included species, describes new Afrotropical, Australian, Oriental and Palearctic species, illustrates all the species through macrophotography, and provides new host and distribution records.

## ﻿Materials and methods

The specimens examined in this study are deposited in the collections of the National Institute of Biological Resources (Incheon, Republic of Korea; **NIBR**), the Science Museum of Natural Enemies (Geochang, Republic of Korea; **SMNE**), G.B.Pant University of Agriculture & Technology (Pantnagar, India; **BUAT**), the Zoological Institute of the Russian Academy of Sciences (St Petersburg, Russia; **ZISP**), the Natural History Museum (London, United Kingdom; **NHMUK**), the Muséum National d’Histoire Naturelle (Paris, France; **MNHN**), the University of California (Riverside, USA; **UCR**); the Canadian National Collection of Insects, Arachnids and Nematodes (Ottawa, Canada; **CNC**), the Institute of Zoology of the Chinese Academy of Sciences (Beijing, China; **IZAS**), the Queensland Museum (Brisbane, Australia: **QMBA**), the National Museum of Natural History, Smithsonian Institution (Washington D.C., USA; **USNM**), Muséum national d’histoire naturelle (Paris, France; **MNHN**), Centre de Biologie pour la Gestion des Populations (Montpellier, France; **CBGP**), Musée royal de l’Afrique centrale / Koninklijk Museum voor Midden-Afrika (Tervuren, Belgium; **MRAC**), and the Mitroiu Collection (Iași, Romania; **MICO**).

Morphological terminology, including sculpture and wing venation, follows Bouček and Rasplus (1991), Gibson (1997), and Burks et al. (2022). The flagellum consists of two anelli, six funicular segments, and the four-segmented clava. The antennal formula includes the number of segments: scape, pedicel, anelli, funicular segments, claval segments. The mandibular formula indicates the number of teeth in the left and right mandibles. The following abbreviations are used: **POL** – posterior ocellar line, the minimum distance between the posterior ocelli; **OOL** – ocello–ocular line, the minimum distance between a posterior ocellus and compound eye; **C1–C4** – claval segments; **M** – marginal vein; **S** – stigmal vein; **PM** – postmarginal vein; **F1–F6** – funicular segments; **Mt2–Mt8** – metasomal tergites. The scape is measured without the radicle; the pedicel is measured in lateral view. The distance between the clypeal lower margin and the toruli is measured from the lower margins of the toruli. The distance between the toruli and the median ocellus is measured from the lower margins of the toruli to the lower margin of the median ocellus. Eye height is measured as the maximum diameter, eye length as the minimum diameter. The mesosoma and metasoma are measured in lateral view, the latter including the ovipositor sheaths.

## ﻿Taxonomic account

### ﻿Class Hexapoda Blainville, 1816

**Order Hymenoptera Linnaeus, 1758**

**Family Pteromalidae Dalman, 1820**

**Subfamily Metasteninae Ashmead, 1904**

#### ﻿Genus Acroclisoides

Taxon classificationAnimaliaHymenopteraPteromalidae

Girault & Dodd, 1915

7FB2F1BF-E8C2-5C65-A891-1CA1ABA3C2CC

Acroclisoides Girault & Dodd, 1915: 344. Type species Acroclisoides megacephalus Girault & Dodd, 1915, by original designation. Type locality Australia, Queensland.
Neocoruna Huang & Liao, 1988: 426; synonymy by Xiao and Huang (2000): 94. Type species: Neocoruna sinica, by original designation. Type locality China, Beijing.
Golovissima Dzhanokmen, 1982. Type species: Golovissima emeljanovi Dzhanokmen, 1982, by original designation. Type locality Australia, Queensland. Syn. nov.

##### Diagnosis.

Head with occipital carina (Figs 7, 15, 24, 40, 103, 111, 142, 150, 174). Gena strongly receding towards mouth, hollowed mouth corner (Figs 6, 22, 30, 38, 46, 62, 70, 78, 86, 94, 102, 110, 125, 133, 141, 149, 157, 165, 181); lower posterior corner of gena either with sharp spine (Figs 46, 62, 70, 78, 86, 94, 141, 149, 181), forming an acute angle (30, 49, 102, 165), or rounded (6, 22, 33, 38, 110, 125, 133, 157). Lower margin of clypeus concave bilaterally (Figs 4, 14, 20, 28, 36, 44, 52, 60, 68, 76, 84, 92, 100, 108, 123, 131, 139, 147, 155, 163, 171, 179), or straight (Fig. 116); in middle part emarginate (Figs 20, 36, 44, 52, 60, 68, 76, 84, 100, 108, 139, 147, 155, 163), weakly rounded (Fig. 131), weakly emarginate or straight (Figs 4, 28, 92, 116, 123, 173, 179). Antennal formula 11264; anelli small, F1–F6 longer than broad, symmetrical, microsetose area small (Figs 3, 11, 19, 27, 35, 43, 51, 59, 67, 75, 83, 91, 99, 107, 115, 122, 130, 138, 146, 154, 162, 170, 178). Antennal toruli situated above level of lower edges of eyes; antennal scrobes deep (Figs 4, 14, 20, 28, 36, 44, 52, 60, 68, 76, 84, 92, 100, 108, 116, 123, 131, 139, 147, 155, 163, 171, 179). Mandibles large, mandibular formula 3:4 or 4:4 (Fig. 44).

Mesosoma short, moderately arched. Pronotum narrower than mesoscutum, with collar margin carinate (Figs 7, 12, 24, 32, 40, 48, 56, 72, 80, 88, 95, 104, 112, 119, 127, 135, 143, 150, 158, 167, 175, 183). Notauli complete (Figs 7, 15, 23, 31, 39, 47, 55, 63, 71, 79, 95, 103, 111, 126, 134, 142, 150, 158, 166, 174, 182). Scutellum arched, without conspicuous sublateral grooves, with weakly distinct frenal area and without frenal groove. Propodeum reticulate, without costula, with distinct median carina (Figs 10, 18, 34, 42, 50, 58, 66, 90, 98, 106, 114, 121, 129, 137, 145, 153, 161, 169, 177) or without (Figs 2, 26, 74, 82), nucha short, propodeal spiracles near to front margin of sclerite. Fore wing hyaline (Figs 5, 13, 45, 53, 61, 77, 85, 109, 117, 124, 132, 172, 180), with one (Figs 29, 37, 69, 93, 101, 140, 148, 156, 164) or two spots (Fig. 21), with closed speculum; M wider than PM. Hind coxa dorsally bare; hind tibia with one spur.

Metasoma shorter than combined length of mesosoma and head, on subconical petiole; Mt2 elongate and narrow; cerci with setae subequal in length; ovipositor not strongly protruding (Figs 7, 15, 23, 31, 39, 47, 55, 63, 71, 79, 87, 95, 103, 111, 118, 126, 134, 142, 150, 158, 166, 174, 182).

##### Distribution.

Nearctic, Palaearctic, Oriental, Afrotropical and Australian regions.

### Key to world species of *Acroclisoides* based on females

**Table:** 

1	Fore wing hyaline (Figs 5, 13, 45, 53, 61, 77, 85, 109, 117, 124, 132, 172, 180)	**2**
–	Fore wing with one (Figs 29, 69, 93, 101, 140, 148, 156, 164) or two spots (Fig. 21), occasionally with diffuse infumation (Fig. 37)	**14**
2	Flagellum with F4–F6 (Fig. 51), F5–F6 (Fig. 170) or F6 yellow or yellowish brown (Figs 11, 83, 115)	**3**
–	Flagellum with F4–F6 brown (Figs 3, 43, 59, 75, 107, 122, 130, 178)	**7**
3	Flagellum with F1–F3 yellow (Fig. 115). Scutellum with smooth frenal area (Figs 114, 119)	***A. quintus* Xiao & Huang**
–	Flagellum with F1–F3 brown or yellowish brown (Figs 11, 51, 83, 170). Scutellum with strongly (Figs 10, 82) or gently (Figs 50, 169) reticulate frenal area	**4**
4	F4–F6 (Fig. 51) or F5–F6 yellow (Fig. 170). Eye height 1.79–1.83 × malar space. Scutellum with finely reticulate frenal area (Figs 50, 169)	**5**
–	F6 yellow (Figs 11, 83). Eye height 1.35–1.46 × malar space. Scutellum with strongly reticulate frenal area (Figs 10, 82)	**6**
5	Clypeus reticulate, distinctly emarginate medially (Fig. 54). Distance between antennal toruli and lower margin of clypeus 2.20–2.50 × distance between antennal toruli and median ocellus. Flagellum with F4–F6 yellow or yellowish-brown (Fig. 51). POL 0.74–0.75 × as long as OOL. Fore and hind coxae dark brown with diffuse blue luster, mid coxae yellow	***A. laticeps* Girault & Dodd**
–	Clypeus striate, straight medially (Fig. 173). Distance between antennal toruli and lower margin of clypeus 3.46 × distance between antennal toruli and median ocellus. Flagellum with F5–F6 yellow (Fig. 170). POL 0.55 × as long as OOL. All coxae yellow	***A. suria* Tselikh, sp. nov.**
6	Clypeus striate (Fig. 14). Flagellum with F1–F2 brown (Fig. 11). Distance between antennal toruli and lower margin of clypeus 2.65–2.74 × distance between antennal toruli and median ocellus. Head in frontal view trapezoidal (Fig. 14)	***A. bicolor* Luo & Qin**
–	Clypeus reticulate (Fig. 84). Flagellum with F1–F2 yellowish brown (Fig. 83). Distance between antennal toruli and lower margin of clypeus 4.50–5.10 × distance between antennal toruli and median ocellus. Head in frontal view oval (Fig. 84)	***A. marimbae* Tselikh & Mitroiu, sp. nov.**
7	Distance between antennal toruli and lower margin of clypeus 4.10–5.00 × distance between antennal toruli and median ocellus. Combined length of pedicel and flagellum 1.12–1.20 × breadth of head. All coxae yellow or yellowish brown	**8**
–	Distance between antennal toruli and lower margin of clypeus 1.58–3.35 × distance between antennal toruli and median ocellus. Combined length of pedicel and flagellum 0.73–1.00 × breadth of head. Fore and hind coxae dark green, mid coxae yellow or yellowish brown	**9**
8	Propodeum without median carina (Fig. 2). Fore wing with small stigma, M 1.35–1.62 × as long as S (Fig. 5). All coxae yellowish brown	***A. africanus* Ferrière**
–	Propodeum with median carina (Fig. 129). Fore wing with large stigma, M as long as S (Fig. 132). All coxae yellow	***A. simbis* Tselikh & Mitroiu, sp. nov.**
9	Lower margin of clypeus deeply concave bilaterally (Fig. 179). Flagellum with F1 1.35–1.50 × as long as broad (Fig. 178)	***A. tectacorisi* (Girault)**
–	Lower margin of clypeus not deeply concave bilaterally (Figs 44, 60, 76, 108, 123). Flagellum with F1 1.65–2.20 × as long as broad (Figs 43, 59, 75, 107, 122)	**10**
10	Lower posterior corner of gena with large spine (Fig. 46). Distance between antennal toruli and lower margin of clypeus 3.14–3.35 × distance between antennal toruli and median ocellus. POL 0.47–0.59 × as long as OOL. Basal cell of fore wing bare or with 1–3 setae (Fig. 45)	***A. indicus* Ferrière**
–	Lower posterior corner of gena with small spine (Figs 62, 78) or rounded (Figs 110, 125). Distance between antennal toruli and lower margin of clypeus 1.83–2.50 × distance between antennal toruli and median ocellus. POL 0.68–0.95 × as long as OOL. Basal cell of fore wing all or partly setose (Figs 61, 77, 109, 124)	**11**
11	Head strongly pubescent (Fig. 123). Scutellum with alutaceous frenal area (Fig. 121). Fore wing with small speculum (Fig. 124)	***A. sativus* Kumar & Khan**
–	Head not strongly pubescent (Figs 60, 76, 108). Scutellum with reticulate frenal area (Figs 58, 74, 106). Fore wing with normal speculum (Figs 61, 77, 109)	**12**
12	POL 0.68–0.70 × as long as OOL. Distance between antennal toruli and lower margin of clypeus 2.43–2.50 × distance between antennal toruli and median ocellus. Fore wing with M longer than S (Fig. 61)	***A. luzonensis* Gahan**
–	POL 0.90–0.95 × as long as OOL. Distance between antennal toruli and lower margin of clypeus 1.92–2.22 × distance between antennal toruli and median ocellus. Fore wing with M shorter than or as long as S (Figs 77, 109)	**13**
13	Clypeus deeply emarginate medially (Fig. 76). Pedicel 0.60–0.65 × as long as F1 (Fig. 75). Head in frontal view oval (Fig. 76). Scutellum with strongly reticulate frenal area (Fig. 74)	***A. major* Girault & Dodd**
–	Clypeus weakly emarginate medially (Fig. 108). Pedicel 0.89–0.90 × as long as F1 (Fig. 107). Head in frontal view trapezoidal (Fig. 108) Scutellum with finely reticulate frenal area (Fig. 106)	***A. nongae* Tselikh, Lee & Ku, sp. nov.**
14	Flagellum with F6 yellow (Figs 138, 146). Stigma of fore wing usually not large (Figs 140, 148)	**15**
–	Flagellum with F1–F6 brown (Figs 19, 27, 35, 67, 91, 99, 154, 162). Stigma of fore wing usually large (Figs 21, 29, 69, 93, 101, 156, 164), except in *A. fusus* (Fig. 37)	**16**
15	Fore wing with M shorter than S and wide (Fig. 148). Frons of head in dorsal view strongly projecting and curved (Fig. 151). Head and mesosoma in dorsal view dark blue (Fig. 150)	***A. solus* Grissell & Smith**
–	Fore wing with M longer than S and less wide (Fig. 140). Frons of head in dorsal view only slightly projecting and curved (Fig. 143). Head and mesosoma in dorsal view bright green with diffuse coppery lustre (Fig. 142)	***A. sinicus* (Huang & Liao)**
16	Fore wing with two spots (Fig. 21). Lower margin of clypeus broadly and deeply concave (Fig. 20)	***A. bimaculatus* Tselikh, sp. nov.**
–	Fore wing with one spot (Figs 29, 37, 69, 93, 101, 156, 164). Lower margin of clypeus not deeply concave (Figs 28, 36, 68, 92, 100, 155, 163)	**17**
17	Lower posterior corner of gena forming an sharp spine (Figs 70, 94). Basal cell of fore wing with 0–7 setae (Figs 69, 93)	**18**
–	Lower posterior corner of gena with acute angle (Figs 30, 102, 165) or rounded (Figs 38, 157). Basal cell of fore wing completely or partly setose (Figs 29, 37, 101, 156, 164)	**19**
18	Clypeus reticulate and distinctly emarginate medially (Fig. 68). Flagellum with F1 1.64–1.83 × as long as broad. Basal cell of fore wing with 0–3 setae (Fig. 69)	***A. maculatus* Sureshan & Narendran**
–	Clypeus striate and weakly emarginate medially (Fig. 92). Flagellum with F1 2.18–2.20 × as long as broad. Basal cell of fore wing with 6 or 7 setae (Fig. 93)	***A. megacephalus* Girault & Dodd**
19	Distance between antennal toruli and lower margin of clypeus 4.50–4.90 × distance between antennal toruli and median ocellus. Antenna with scape 1.18–1.19 × as long as eye length. Stigma of fore wing not large (Fig. 37)	***A. fusus* Tselikh & Mitroiu, sp. nov.**
–	Distance between antennal toruli and lower margin of clypeus 1.77–2.57 × distance between antennal toruli and median ocellus. Antenna with scape 0.87–1.08 × as long as eye length. Stigma of fore wing large (Figs 29, 101, 156, 164)	**20**
20	Flagellum with F1 1.60–1.80 × as long as broad (Fig. 27). Propodeum without distinct median carina (Fig. 26). Scutellum with alutaceous frenal area (Fig. 26)	***A. emeljanovi* Dzhanokmen**
–	Flagellum with F1 2.00–2.80 × as long as broad (Figs 99, 154, 162). Propodeum with distinct median carina (Figs 98, 153, 161). Scutellum with reticulate frenal area (Figs 98, 153, 161)	**21**
21	Clypeus reticulate (Fig. 155). Distance between antennal toruli and lower margin of clypeus 2.44–2.57 × distance between antennal toruli and median ocellus. Scutellum with finely reticulate frenal area (Fig. 153)	***A. spilopterus* (Masi)**
–	Clypeus striate-reticulate or striate (Figs 100, 163). Distance between antennal toruli and lower margin of clypeus 1.77–2.30 × distance between antennal toruli and median ocellus. Scutellum with distinct reticulate frenal area (Figs 98, 161)	**22**
22	Distance between antennal toruli and lower margin of clypeus 2.27–2.30 × distance between antennal toruli and median ocellus. Stigma of fore wing 0.65–0.70 × as long as high (Fig. 164). Middle part of clypeus reticulate-striate (Fig. 163)	***A. supramaculatus* Tselikh, sp. nov.**
–	Distance between antennal toruli and lower margin of clypeus 1.77–1.78 × distance between antennal toruli and median ocellus. Stigma of fore wing 1.28–1.33 × as long as high (Fig. 101). Middle part of clypeus striate (Fig. 101)	***A. miklukhai* Tselikh, sp. nov.**

#### ﻿ Acroclisoides africanus

Taxon classificationAnimaliaHymenopteraPteromalidae

Ferrière, 1940

15F54960-0312-570D-8EC5-622F38CDA652

Figs 1–8

Acroclisoides africanus Ferrière, 1940: 145. Syntype female (NHMUK, examined).

##### Type material.

***Syntype*** • female, Kenya, “E. AFRICA Kenya”, “1137. 17.7.33 Ex Eggs *Antestia lineaticollis* Kiambu R.H. LePelley”, “1137”, “Type”, “*Acroclisoides africanus* ♀ Ch. Ferrière Type”, “Pres. By Imp. Inst. Ent. B.M. 1940-163”, “B.M. TYPE HYM. 5.865” (NHMUK).

**Figures 1–8.**
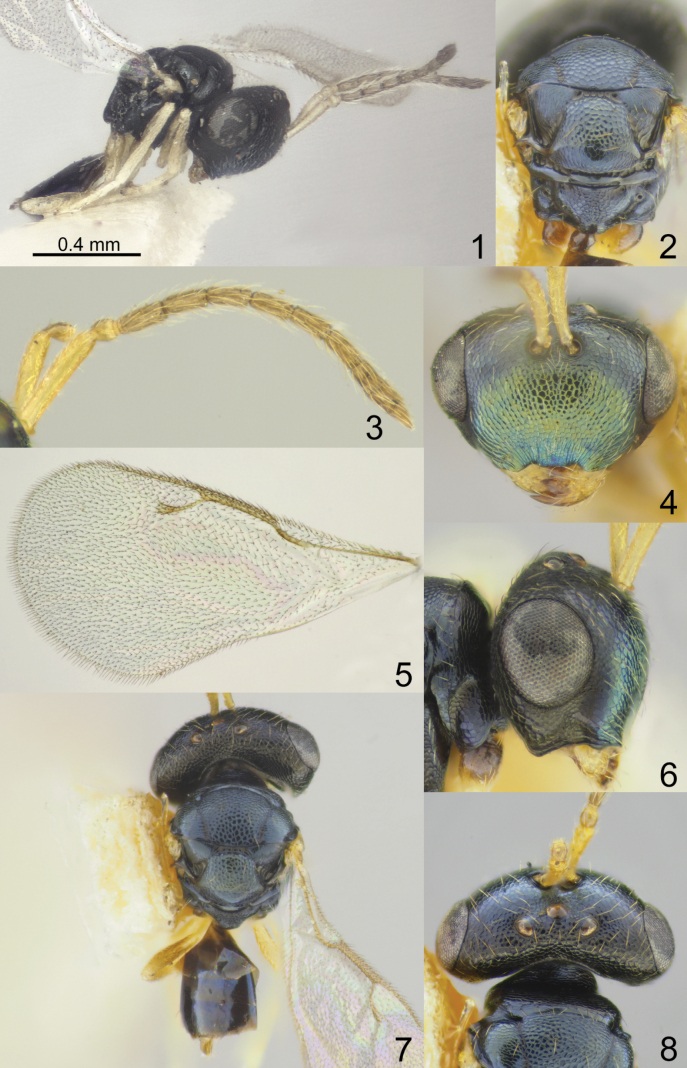
*Acroclisoides africanus* Ferrière, 1940, 1. female, holotype and 2–8. female, not type. 1. Habitus, lateral view; 2. Mesosoma and propodeum, dorsal view; 3. Antenna; 4. Head, frontal view; 5. Fore wing; 6. Head, lateral view; 7. Habitus, dorsal view; 8. Head and pronotum, dorsal view.

##### Additional material examined.

Democratic Republic of the Congo • 5 females, “BELGIAN CONGO Mulungu Dist. 1947 P.C. Lefevre 492”, “IMP. INST ENT. COLL. NO. 10426”, “Ex. *Antestia* sp. nr. *Lineaticollis* Stal.”, “Pres. By Imp. Inst. Ent. B.M. 1948-321”, “NHMUK 013456011”, “NHMUK 013456015”, “NHMUK 013456016”, “NHMUK 013456017”, “NHMUK 013456018” (NHMUK, ZISP) • 2 females, “Mus. Congo, Rutshuru, III-1937, J. Ghesquière, 6135” (MRAC) • 1 female, “Mus. Congo, Mulungu, 1937, P. Lefèvre, 27”, “Récolté sur Caféier” (MRAC). Uganda • 8 females, 4 males, “UGANDA Jono 5.IV.1926 H. Hargreaves”, “Ex eggs *Antestia lineaticollis*”, “NHMUK 013455892”, “NHMUK 013455893”, “NHMUK 013455894”, “NHMUK 013455895”, “NHMUK 013455896”, “NHMUK 013455897”, “NHMUK 013455898”, “NHMUK 013455899”, “NHMUK 013455900”, “NHMUK 013455901”, “NHMUK 013455902”, “NHMUK 013455903” (NHMUK).

##### Description.

**Female.** Body length 1.13–1.50 mm; fore wing length 1.13–1.45 mm.

***Coloration*.** Head and mesosoma dorsally dark blue with diffuse metallic luster; head frontally dark green with diffuse coppery luster; antenna with scape yellow; pedicel and anelli yellowish brown; F1–F6 and clava brown. All coxae, femora and tibiae yellowish brown, all tarsi yellow. Fore wing hyaline, venation brown. Metasoma dorsally dark brown with metallic blue luster; ovipositor sheaths yellowish brown or brown.

***Sculpture*.** Head and mesosoma reticulate; clypeus striate-reticulate; scutellum strongly reticulate, but frenal area finely reticulate; propodeum reticulate, nucha alutaceous; petiole smooth; metasoma smooth and shiny.

***Head*.** In dorsal view 2.10–2.22 × as broad as long and 1.15–1.38 × as broad as mesoscutum; in frontal view 1.40–1.50 × as broad as high. POL 0.75–0.90 × as long as OOL. Eye height 1.11–1.19 × eye length and 1.66–1.82 × malar space. Distance between antennal toruli and lower margin of clypeus 4.70–5.00 × distance between antennal toruli and median ocellus. Antenna with scape 0.97–1.00 × as long as eye height and 1.11–1.15 × as long as eye length; pedicel 1.25–1.38 × as long as broad; combined length of pedicel and flagellum 1.12–1.20 × breadth of head; F1–F6 longer than broad, F1 2.10–2.66 × as long as broad and with two rows of sensilla; clava 2.80–3.00 × as long as broad, with small microsetose area on C3 and C4. Lower posterior corner of gena rounded. Lower margin of clypeus deeply concave bilaterally, in middle part arched and straight.

***Mesosoma*.** 1.20–1.26 × as long as broad. Scutellum moderately arched, 0.83–0.85 × as long as broad, frenal area differentiated by a change in sculpture. Propodeum 0.50–0.54 × as long as scutellum, without costula and median carina, nucha small. Fore wing 1.89–2.06 × as long as its maximum width; basal cell setose; basal vein setose; speculum small and closed below; M 0.76–0.81 × as long as PM and 1.35–1.62 × as long as S, stigma small.

***Metasoma*.** 1.39–1.40 × as long as broad, 0.83–0.89 × as long as mesosoma, 0.61–0.72 × as long as mesosoma and head. Petiole 1.00–1.22 × as long as broad. Ovipositor sheaths projecting slightly beyond apex of metasoma.

**Male.** Body length 0.85–0.92 mm; fore wing length 0.75–0.90 mm. Distance between antennal toruli and lower margin of clypeus 3.00–3.10 × distance between antennal toruli and median ocellus. Propodeum 0.70–0.76 × as long as scutellum. Fore wing with M 0.95–1.00 × as long as PM. Otherwise, similar to female.

##### Biology.

Egg parasitoid of the hemipterans *Antestiopsis faceta* (Germar, 1837), *A. lineaticollis* (Stål, 1853), *A. facetoides* Greathead, 1965, *A. intricata* (Ghesquière & Carayon, 1948), *A. orbitalis* (Westwood, 1837), *A. thunbergii* (Gmelin, 1790), *Atelocera notatipennis* Stål, 1858, (Pentatomidae) and hyperparasitoids of *Asolcus* sp. and *Trissolcus* sp. (Scelionidae) (UCD Community 2025).

##### Distribution.

Burundi, Democratic Republic of the Congo, Kenya, Madagascar, Senegal, Uganda (UCD Community 2025).

##### Comments.

*Acroclisoides africanus* Ferrière belongs to a group of species where both sexes have a hyaline fore wing. This species is very similar to *A. simbis* Tselikh & Mitroiu, sp. nov.; the differences between these species are given in the key.

Material from Ghana was misidentified as *A. africanus* Ferrière by D.S. Hill; in this paper this material is considered to be a new species, *A. fusus* Tselikh & Mitroiu, sp. nov. (see below).

#### ﻿ Acroclisoides bicolor

Taxon classificationAnimaliaHymenopteraPteromalidae

Luo & Qin, 1991

0D7B027E-00E3-55CC-B50D-8B5DF17BC39D

Figs 9–16

Acroclisoides bicolor Luo & Qin, 1991: 364, 365. Holotype female (IZAS, not examined, not found in collection).

##### Material examined.

Russia • 1 female, “Primorskii Reg., Spassk, 23.VIII.1987, coll. S. Belokobylskij” (ZISP). China • 1 female, 1 male, “Jiangxi 1978.VII.13”, “Liao, Dingxi” (IZAS) • 2 males, “vii 1973 HUNAN Changsha”, “Erthesina fullo D.X. Liao” (IZAS).

**Figures 9–16.**
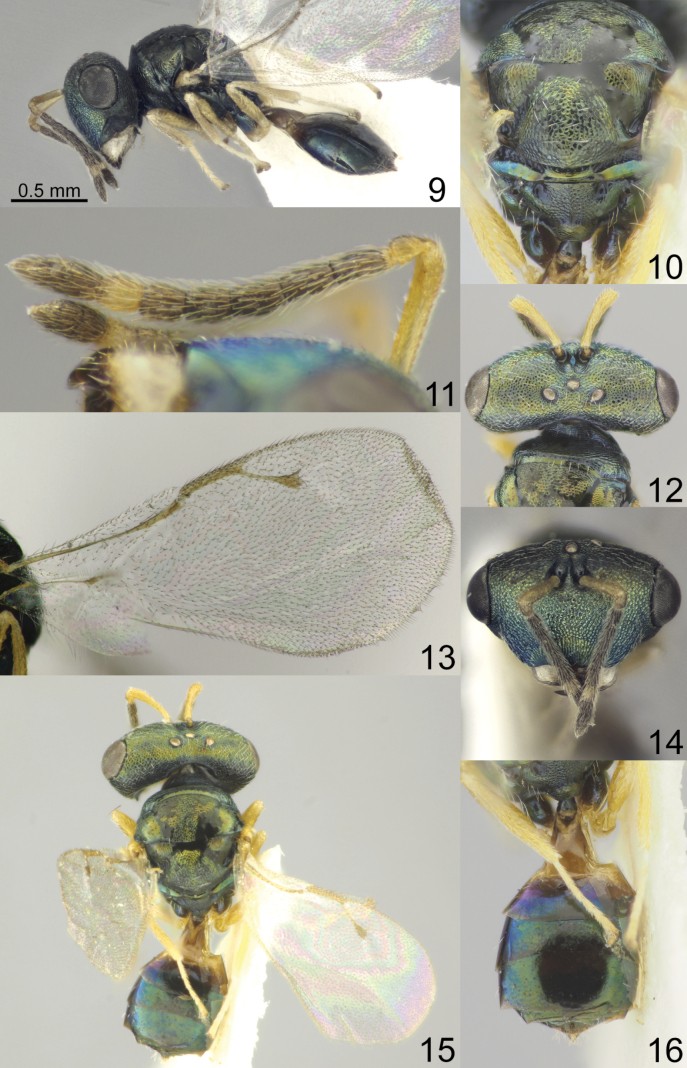
*Acroclisoides bicolor* Luo & Qin, 1991, female, not type. 9. Habitus, lateral view; 10. Mesosoma and propodeum, dorsal view; 11. Antenna; 12. Head and pronotum, dorsal view; 13. Fore wing; 14. Head, frontal view; 15. Habitus, dorsal view; 16. Metasoma, dorsal view.

##### Description.

**Female.** Body length 1.85–2.30 mm; fore wing length 1.70–2.20 mm.

***Coloration*.** Head and mesosoma dark green blue with diffuse coppery luster; antenna with scape, pedicel and anelli yellowish brown; F1–F5 and clava brown, F6 yellow. Fore and hind coxa dark green with diffuse coppery luster, mid coxae yellow, all femora and tibiae yellowish brown, all tarsi yellow. Fore wing hyaline, venation brown. Metasoma dorsally dark green with diffuse coppery and violet luster; ovipositor sheaths yellowish brown or brown.

***Sculpture*.** Head and mesosoma reticulate; clypeus striate; scutellum and propodeum reticulate, nucha alutaceous; petiole smooth; metasoma smooth and shiny.

***Head*.** In dorsal view 2.30–2.50 × as broad as long and 1.40–1.43 × as broad as mesoscutum; in frontal view 1.63–1.70 × as broad as high. POL 0.61–0.70 × as long as OOL. Eye height 1.13–1.20 × eye length and 1.35–1.42 × malar space. Distance between antennal toruli and lower margin of clypeus 2.65–2.74 × distance between antennal toruli and median ocellus. Antenna with scape 0.94–1.05 × as long as eye height and 1.06–1.15 × as long as eye length; pedicel 1.32–1.38 × as long as broad; combined length of pedicel and flagellum 0.75–0.78 × breadth of head; F1–F6 longer than broad, F1 1.85–1.90 × as long as broad and with two rows of sensilla; clava 2.40–2.70 × as long as broad, with small microsetose area on C3 and C4. Lower posterior corner of gena rounded. Lower margin of clypeus not deeply concave bilaterally, in middle part weakly emarginate.

***Mesosoma*.** 1.20–1.22 × as long as broad. Scutellum moderately arched, 0.73–0.86 × as long as broad, frenal area differentiated by a change in sculpture. Propodeum 0.65–0.70 × as long as scutellum, without costula but with median carina, nucha small. Fore wing 2.00 × as long as its maximum width; basal cell setose; basal vein setose; speculum small and closed below; M 0.75–0.76 × as long as PM and 1.00–1.13 × as long as S, stigma small.

***Metasoma*.** 1.60–1.95 × as long as broad, 1.04–1.09 × as long as mesosoma, 0.80–0.83 × as long as mesosoma and head. Petiole 1.00–1.11 × as long as broad. Ovipositor sheaths projecting slightly beyond apex of metasoma.

**Male.** Body length 1.53–1.70 mm; fore wing length 1.40–1.65 mm. Combined length of pedicel and flagellum 0.88–0.95 × breadth of head. F1-F5 yellowish brown. Otherwise, similar to female.

##### Biology.

Unknown.

##### Distribution.

Russian Far East, China (Luo and Qin 1991; Tselikh 2016).

##### Comments.

*Acroclisoides bicolor* Luo & Qin belongs to a group of species where both sexes have a hyaline fore wing. This species is very similar to *A. marimbae* Tselikh & Mitroiu, sp. nov.; the differences between these species are given in the key. It is also similar to *A. sinicus* (Huang & Liao) and *A. solus* Grissel & Smith, from which it differs in having the fore wing hyaline (although some female specimens of *A. solus* may also have hyaline wings, see the comments under *A. solus*) and the posterior corner of gena rounded.

Material from China was misidentified as *A. indicus* Ferrière. Here this material is regarded as *A. bicolor* Luo & Qin, 1991.

#### ﻿ Acroclisoides bimaculatus

Taxon classificationAnimaliaHymenopteraPteromalidae

Tselikh sp. nov.

D062BB65-31F9-500F-AA95-EF8B27AAF8AB

https://zoobank.org/7A3FCBF5-F61D-4CE5-B031-E3F57C1A0F44

Figs 17–24

##### Type material.

***Holotype*** • female, Australia, “AUS. C.T. Canberra Black Mtn. II. 1975 Z. Liepa”, “Ex eggs coll. 7 Feb. 1975”, “NHMUK 013455911” (NHMUK).

##### Diagnosis.

*Acroclisoides bimaculatus* Tselikh, sp. nov. belongs to a group of species where females have maculated fore wings, but this species can be easily distinguished from the others in having the fore wings with two spots (Fig. 21) and a broadly deeply concave lower margin of the clypeus (Fig. 20).

**Figures 17–24.**
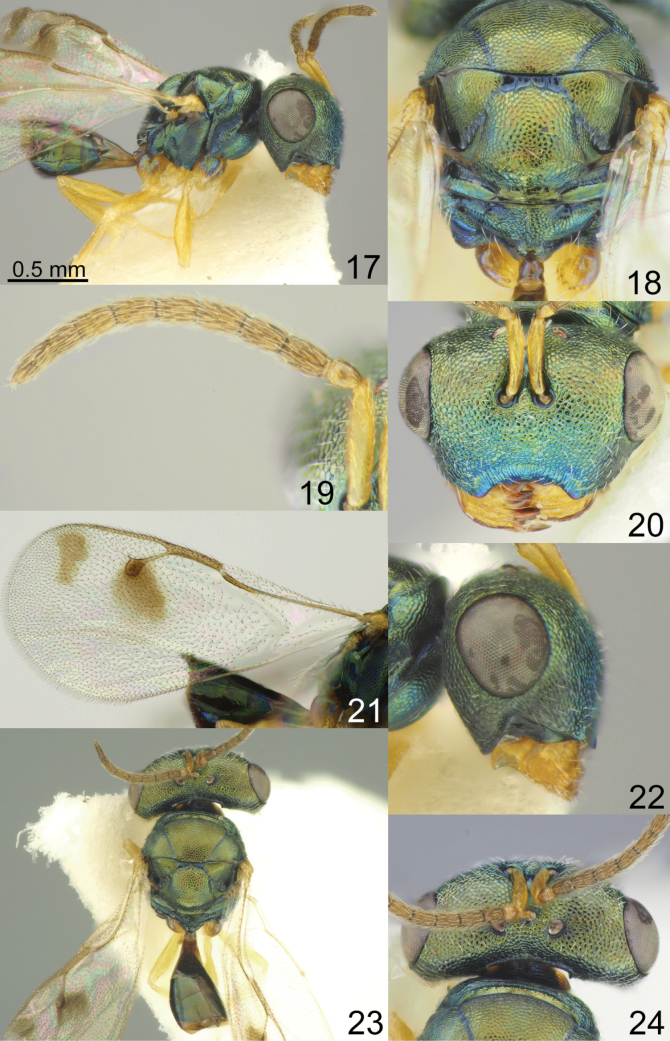
*Acroclisoides bimaculatus* Tselikh, sp. nov., female, holotype. 17. Habitus, lateral view; 18. Mesosoma and propodeum, dorsal view; 19. Antenna; 20. Head, frontal view; 21. Fore wing; 22. Head, lateral view; 23. Habitus, dorsal view; 24. Head and pronotum, dorsal view.

##### Description.

**Female.** Body length 2.10 mm; fore wing length 2.00 mm.

***Coloration*.** Head and mesosoma dark green blue with diffuse coppery luster; antenna with scape yellow, pedicel dorsally brown, ventrally yellowish brown; anelli yellowish brown; F1–F6 and clava brown. Fore and hind coxa dark green with diffuse coppery luster, mid coxae yellow, all femora, tibiae and tarsi yellow. Fore wing with two spots, venation brown. Metasoma dorsally dark brown with metallic blue-green and coppery luster; ovipositor sheaths brown.

***Sculpture*.** Head and mesosoma reticulate; clypeus striate, but small middle part reticulate; scutellum and propodeum reticulate, nucha alutaceous; petiole smooth; metasoma smooth and shiny.

***Head*.** In dorsal view 2.20 × as broad as long and 1.39 × as broad as mesoscutum; in frontal view 1.58 × as broad as high. POL 0.62 × as long as OOL. Eye height 1.14 × eye length and 2.16 × malar space. Distance between antennal toruli and lower margin of clypeus 2.00 × distance between antennal toruli and median ocellus. Antenna with scape 0.83 × as long as eye height and 0.94 × as long as eye length; pedicel 1.25 × as long as broad; combined length of pedicel and flagellum 0.85 × breadth of head; F1–F6 longer than broad, F1 1.58 × as long as broad and with two rows of sensilla; clava 2.46 × as long as broad, with small microsetose area on C3 and C4. Lower posterior corner of gena rounded. Lower margin of clypeus broadly deeply concave, in middle part weakly emarginate.

***Mesosoma*.** 1.23 × as long as broad. Scutellum moderately arched, 0.75 × as long as broad, frenal area differentiated by a change in sculpture. Propodeum 0.67 × as long as scutellum, without costula and with median carina, nucha small. Fore wing 2.10 × as long as its maximum width; basal cell with 2–6 setae; basal vein setose; speculum closed below; M 0.72 × as long as PM and 1.13 × as long as S, stigma large.

***Metasoma*.** 1.91 × as long as broad, 0.83 × as long as mesosoma, 0.56 × as long as mesosoma and head. Petiole 1.11 × as long as broad. Ovipositor sheaths projecting slightly beyond apex of metasoma.

**Male.** Unknown.

##### Etymology.

From the Latin *bis* and *macula*, referring to the two spots on fore wing of this species (adjective).

##### Biology.

Unknown.

##### Distribution.

Australia.

#### ﻿ Acroclisoides emeljanovi

Taxon classificationAnimaliaHymenopteraPteromalidae

(Dzhanokmen, 1982) comb. nov.

43D1BAD5-2AA5-587C-8E0C-247E98B2F815

Figs 25–32

Golovissima emeljanovi Dzhanokmen, 1982: 1600–1601. Holotype female (ZISP, examined).

##### Type material examined.

***Holotype*** • female, Russia, “Primorskii Reg., Anisimovka 14–19.VII.72 Kuslitskij”, “Holotypus *Golovissima emeljanovi* Dzhanokmen, 982” (ZISP). ***Paratypes*** • 11 females, 3 males, Russia, same labels as holotype (ZISP).

**Figures 25–32.**
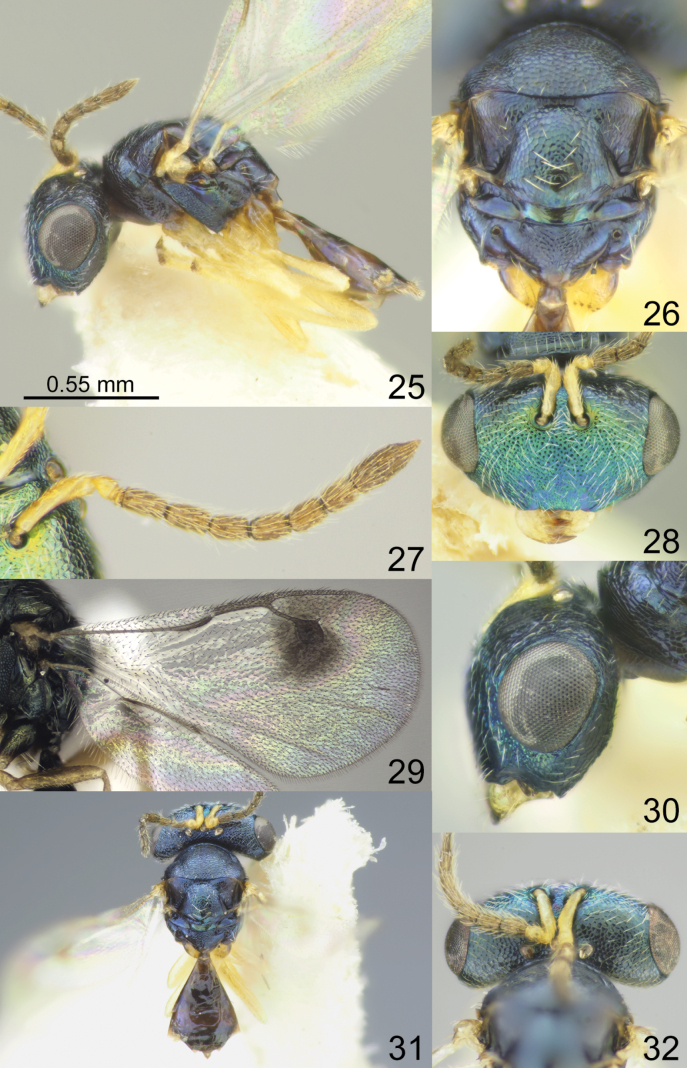
*Acroclisoides emeljanovi* Dzhanokmen, 1982, female, holotype. 25. Habitus, lateral view; 26. Mesosoma and propodeum, dorsal view; 27. Antenna; 28. Head, frontal view; 29. Fore wing; 30. Head, lateral view; 31. Habitus, dorsal view; 32. Head and pronotum, dorsal view.

##### Additional material examined.

Russia • 1 female, “Vladivostok, Shamora, 31.VIII.961 Nikol’skaya” (ZISP) • 1 female, “Vladivostok, 24 km Lyanchikhe, 29.VII.963 Kerzhner” (ZISP) • 1 female, “Vladivostok, Lazurnaya, 15.VIII.978 Kasparyan” (ZISP) • 1 female, “Gorno-Tayozhnaya, 20 km SE Ussuriysk, 29.VIII.978 Kasparyan” (ZISP) • 4 females, “Primorskii Reg., Spassk, 19.VIII.1987, 13.IX.1988, 7.VI.1989, Belokobylskij” (ZISP) • 5 females, “Primorskii Reg., Anisimovka, 4.IX.1988, 6.VIII.2010, Belokobylskij” (NHMUK, ZISP) • 1 female, “Primorskii Reg., 15 km NW Artem, 7.IX.1988, Belokobylskij” (ZISP) • 1 female, “Primorskii Reg., 10 km E Spassk, 11.IX.1988, Belokobylskij” (ZISP) • 1 female, “Primorskii Reg., 20 km NW Spassk, 23.IX.1988, Belokobylskij” (ZISP) • 3 females, “Primorskii Reg., Ussurijsky Reserve, Kamenushka-Kaymanovka, 31.VII–7.VIII.2008, coll Khalaim” (ZISP) • 2 females, “Primorskii Reg., Lazovsky Reserve, Preobrazhenie, 16.VIII.2010, coll. Tselikh, Rachin” (ZISP) • 1 female, “Primorskii Reg., Kondratenovka, 27.VIII.2010, coll. Tselikh, Rachin” (ZISP) • 3 females, “Amur Reg., Khingansky Reserve, Kundur, 17–20.VII.2003, coll. Belokobylskij” (ZISP) • 1 female, “Amur Reg., Khingansky Reserve, 3 km E Uril, 3–4.VIII.2022, coll. Kosheleva” (ZISP). Republic of Korea • 1 female, “Gyeongsangbuk-do, Yeongyang-gun, Irwol-myeon, 36°48'29"N, 129°05'25"E, 14.VII.2015, coll. Tselikh” (ZISP) • 1 female, “S. Korea, Gyeongsangbuk-do, JuWangSan-myeon, Cheongsong-gun, (Mt), JuWangSan, 12.V.2022, coll. J.H. Lee” (SMNE).

##### Description.

**Female.** Body length 2.40 mm; fore wing length 2.65 mm.

***Coloration*.** Head and mesosoma dorsally dark blue with diffuse metallic violet luster; head frontally dark green with diffuse coppery luster; antenna with scape, pedicel and anelli yellow, F1–F6 and clava brown. Fore and hind coxae basally brown with metallic blue luster, apically yellowish brown, mid coxae yellowish brown; all femora, tibiae and tarsi yellow. Fore wing with one spot near S, venation brown. Metasoma dorsally dark brown with metallic blue and violet luster; ovipositor sheaths brown.

***Sculpture*.** Head and mesosoma reticulate; clypeus reticulate-striate; scutellum strongly reticulate, but frenal area alutaceous; propodeum reticulate, nucha smooth; petiole smooth; metasoma smooth and shiny.

***Head*.** In dorsal view 2.52–2.95 × as broad as long and 1.51–1.56 × as broad as mesoscutum; in frontal view 1.67–1.68 × as broad as high. POL 0.81–0.82 × as long as OOL. Eye height 1.13–1.17 × eye length and 1.54–1.78 × malar space. Distance between antennal toruli and lower margin of clypeus 2.00–2.30 × distance between antennal toruli and median ocellus. Antenna with scape 0.75–0.76 × as long as eye height and 0.87–0.89 × as long as eye length; pedicel 1.20–1.25 × as long as broad; combined length of pedicel and flagellum 0.91–0.92 × breadth of head; F1–F6 longer than broad, F1 1.60–1.80 × as long as broad and with one or two rows of sensilla; clava 2.53–3.40 × as long as broad, with small microsetose area on C3 and C4. Lower posterior corner of gena forming an acute angle. Lower margin of clypeus shallowly concave bilaterally, in middle part weakly emarginate.

***Mesosoma*.** 1.34–1.40 × as long as broad. Scutellum moderately arched, 0.83–0.85 × as long as broad, frenal area differentiated by a change in sculpture. Propodeum 0.70–0.73 × as long as scutellum, without costula or median carina, nucha small. Fore wing 1.95–2.00 × as long as its maximum width; basal cell and basal vein setose; speculum small and closed below; M 0.68–0.83 × as long as PM and 0.85–1.00 × as long as S, stigma large.

***Metasoma*.** 1.48–1.55 × as long as broad, 0.92–0.93 × as long as mesosoma, 0.65–0.70 × as long as mesosoma and head. Petiole 1.00–1.11 × as long as broad. Ovipositor sheaths projecting slightly beyond apex of metasoma.

**Male.** Body length 1.50–1.65 mm; fore wing length 1.45–1.60 mm. Combined length of pedicel and flagellum 0.98–1.10 × breadth of head. Fore wing with M 0.95–1.05 × as long as PM. Metasoma 1.85–2.08 × as long as broad. Otherwise, similar to female.

##### Biology.

Egg parasitoids of the hemipteran *Acanthosoma* sp. (Acanthosomatidae) (Dzhanokmen 1982).

##### Distribution.

Republic of Korea, Russian Far East (Dzhanokmen 1982; Lee et al. 2019).

##### Comments.

*Acroclisoides emeljanovi* (Dzhanokmen) belongs to a group of species where females have a fore wing with one spot. This species is similar to *A. spilopterus* (Masi), *A. supramaculatus* Tselikh, sp. nov. and *A. miklukhai* Tselikh, sp. nov.; the differences between these species are given in the key.

#### ﻿ Acroclisoides fusus

Taxon classificationAnimaliaHymenopteraPteromalidae

Tselikh & Mitroiu sp. nov.

1C72B334-4286-5481-85A8-1CE8DF627950

https://zoobank.org/56794011-4B1B-44FA-9AB4-C9213F4B4CD6

Figs 33–40

##### Type material.

***Holotype*** • female, Ghana, “Ghana, Tafo 26.IV.66 Ex. *Bathycoelia thalassina* C.I.E.A. 982”, “*Acroclisoides africanus* Ferrière D.S. Hill det. 1966”, “NHMUK 013455986” (NHMUK). ***Paratypes*** • 2 females, Cameroon, “Cameroon: Nkoemuon, 24.viii–7.ix.1980, D. Jackson” (NHMUK) • 1 female, Cameroon, “Cameroon: Nkoemuon, 13.vii–24.viii.1980, D. Jackson” (MICO) • 3 females, 1 male, Ghana, same data as holotype, “NHMUK 013455981”, “NHMUK 013455985” (NHMUK) • 4 females, Ghana, “Ghana, Tafo 21.II.66 Ex. *Atelocera* C.I.E.A. 982”, “*Acroclisoides africanus* Ferrière D.S. Hill det. 1966”, “NHMUK 013455980”, “NHMUK 013455984” (NHMUK, ZISP).

**Figures 33–40.**
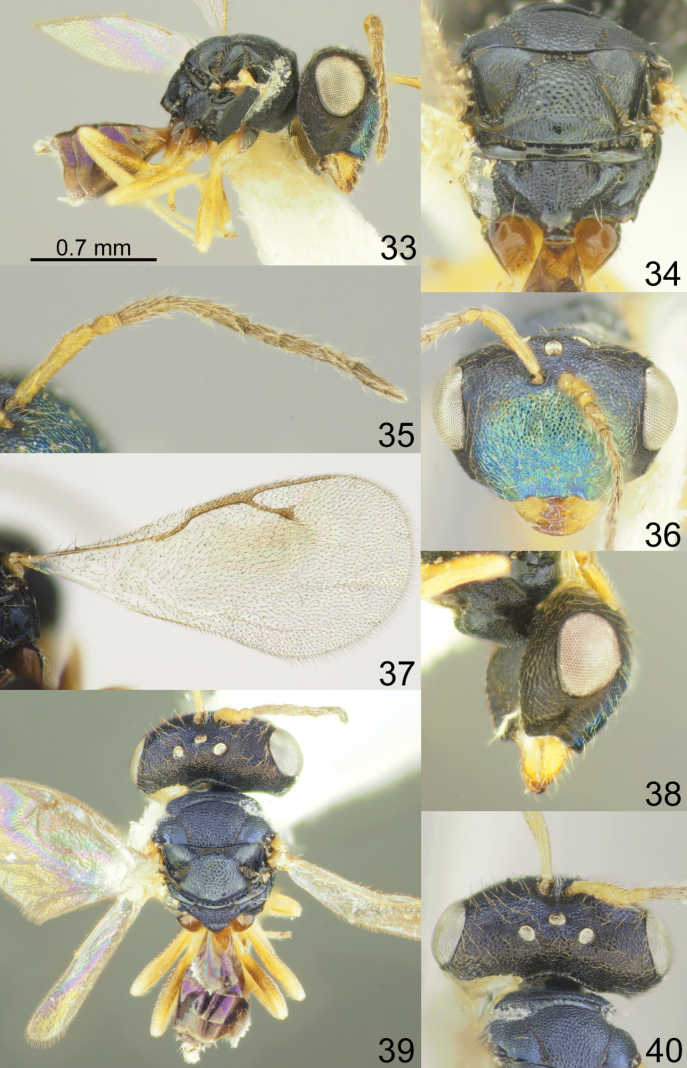
*Acroclisoides fusus* Tselikh & Mitroiu, sp. nov., female, holotype. 33. Habitus, lateral view; 34. Mesosoma and propodeum, dorsal view; 35. Antenna; 36. Head, frontal view; 37. Fore wing; 38. Head, lateral view; 39. Habitus, dorsal view; 40. Head and pronotum, dorsal view.

##### Diagnosis.

*Acroclisoides fusus* Tselikh & Mitroiu, sp. nov. belongs to a group of species where females have a fore wing with one spot, but this species is easily distinguished from the others in having the fore wing with a small stigma (Fig. 37).

##### Description.

**Female.** Body length 1.80–2.20 mm; fore wing length 1.60–1.70 mm.

***Coloration*.** Head and mesosoma dorsally dark blue with diffuse metallic violet luster; head frontally dark blue green with diffuse coppery luster; antenna with scape, pedicel and anelli yellowish brown, F1–F6 and clava brown. All coxae basally brown with metallic blue luster, apically yellowish brown; all femora yellowish brown, tibiae and tarsi yellow. Fore wing with one diffuse spot near S, venation brown. Metasoma dorsally dark brown with metallic violet luster; ovipositor sheaths brown.

***Sculpture*.** Head and mesosoma reticulate; clypeus reticulate-striate; scutellum strongly reticulate, but frenal area finely reticulate; propodeum reticulate, nucha smooth; petiole smooth; metasoma smooth and shiny.

***Head*.** In dorsal view 2.15–2.19 × as broad as long and 1.47–1.48 × as broad as mesoscutum; in frontal view 1.47–1.54 × as broad as high. POL 0.74–0.80 × as long as OOL. Eye height 1.18–1.23 × eye length and 1.46–1.61 × malar space. Distance between antennal toruli and lower margin of clypeus 4.50–4.90 × distance between antennal toruli and median ocellus. Antenna with scape 0.95–1.00 × as long as eye height and 1.18–1.19 × as long as eye length; pedicel 1.00–1.33 × as long as broad; combined length of pedicel and flagellum 1.05–1.14 × breadth of head; F1–F6 longer than broad, F1 2.16–2.20 × as long as broad and with two or three rows of sensilla; clava 3.00–3.33 × as long as broad, with small microsetose area on C3 and C4. Lower posterior corner of gena rounded. Lower margin of clypeus concave bilaterally, in middle part arched and emarginate.

***Mesosoma*.** 1.21–1.22 × as long as broad. Scutellum moderately arched, 0.74–0.77 × as long as broad, frenal area differentiated by a change in sculpture. Propodeum 0.72–0.76 × as long as scutellum, without costula but with median carina, nucha not small. Fore wing 2.13–2.18 × as long as its maximum width; basal cell and basal vein setose; speculum small and closed below; M 0.70–0.75 × as long as PM and 1.29–1.42 × as long as S, stigma small.

***Metasoma*.** 2.04–2.28 × as long as broad, 1.04–1.15 × as long as mesosoma, 0.71–0.81 × as long as mesosoma and head. Petiole 1.00–1.20 × as long as broad. Ovipositor sheaths projecting slightly beyond apex of metasoma.

**Male.** Body length 1.80 mm; fore wing length 1.70 mm. F1 3.25 × as long as broad. Combined length of pedicel and flagellum 1.20 × breadth of head. Fore wing with M 1.40 × as long as S. Otherwise, similar to female.

##### Etymology.

The name of the species is based on the Latin word *fusus* meaning broad or diffuse, referring to the brown spot of the fore wing (adjective).

##### Biology.

Egg parasitoid of hemipterans *Bathycoelia thalassina* (Herrich-Schäffer, 1844) and *Atelocera* sp. (Pentatomidae).

##### Distribution.

Cameroon, Ghana.

##### Comments.

It is important to note that the material from Ghana was misidentified as *A. africanus* Ferrière by D.S. Hill. Here this material is regarded as *A. fusus* Tselikh & Mitroiu, sp. nov., because it can be distinguished from *A. africanus* by the fore wing with one spot near S (vs hyaline); propodeum with median carina and large and smooth nucha (vs propodeum without median carina and with small, alutaceous nucha); lower margin of clypeus in middle part arched and emarginate (vs arched and straight).

#### ﻿ Acroclisoides indicus

Taxon classificationAnimaliaHymenopteraPteromalidae

Ferrière, 1931

24A1D81D-8732-57D1-B6B8-A8723F0978E3

Figs 41–48

Acroclisoides indicus Ferrière, 1931: 279. Holotype female (NHMUK, not examined).

##### Type material examined.

***Paratypes*** • 1 female, India, “Dehra Dun, U.P.S.N. Chatterjee 19.XI.1927”, “767”, “Ex Pentatomidae eggs.”, “On Teak leaf.”, “Pres. By Imp. Inst. Ent. B.M. 1931-367”, “*Acroclisoides indicus* sp. n. Ch. Ferrière det. ♂”, “♀”, “Paratype”, “NHMUK 013455990” (NHMUK) • 1 female, India, “Dehra Dun, U.P.S.N. Chatterjee 19.XI.1927”, “770”, “Ex Pentatomidae eggs.”, “On Teak leaf.”, “Pres. By Imp. Inst. Ent. B.M. 1931-367”, “*Acroclisoides indicus* sp. n. Ch. Ferrière det. ♂”, “♀ det. Tselikh, 2014”, “Paratype”, “NHMUK(E) 953698”, “NHMUK 013455990” (NHMUK) • 2 females, “INDIA: Madras XII.1974 no. 4 Anantakrishnan” “NHMUK 013455886” (NHMUK).

**Figures 41–48.**
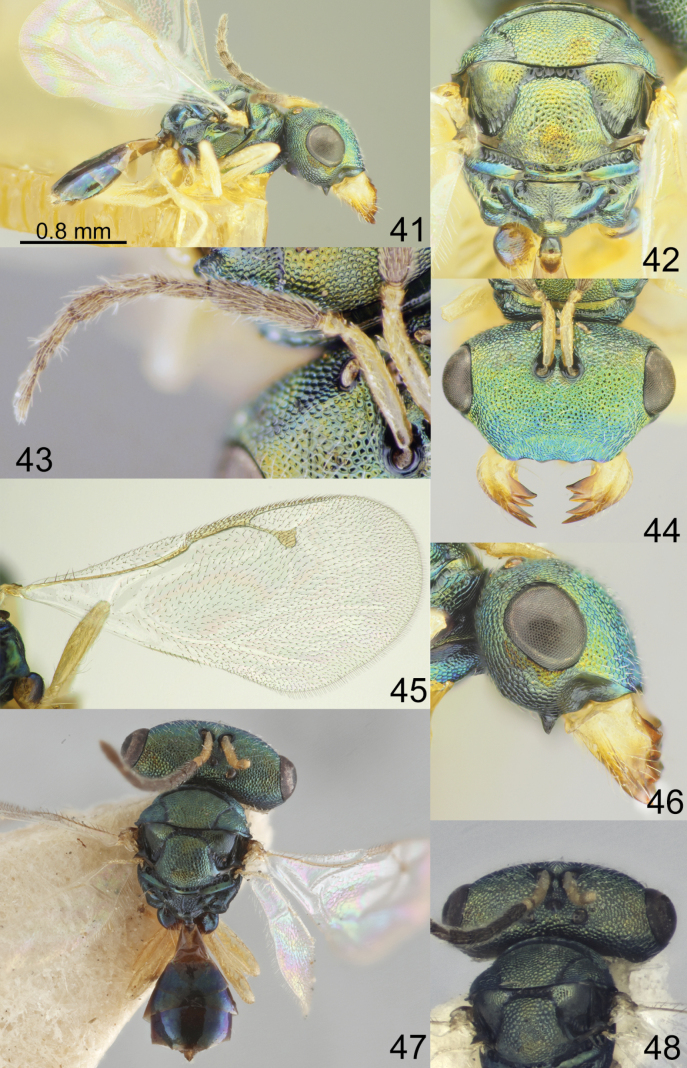
*Acroclisoides indicus* Ferrière, 1931. 47, 48. Female, paratype and 41–46. Female, not type. 41. Habitus, lateral view; 42. Mesosoma and propodeum, dorsal view; 43. Antenna; 44. Head, frontal view; 45. Fore wing; 46. Head, lateral view. 47. Habitus, dorsal view; 48. Head and pronotum, dorsal view.

##### Additional material examined.

Myanmar • 1 female, “Gwethe Res. N. Toungoo. M.H. Desai coll. 20.XII.1934”, “*Acroclisoides indicus* Ferr. Ch. Ferrière det.”, “216”, “Pres. By Imp. Inst. Ent. B.M. 1937-132”, “R.R.S.NO. 1423”, “NHMUK 013455993” (NHMUK).

##### Description.

**Female.** Body length 2.06–2.80 mm; fore wing length 1.90–2.30 mm.

***Coloration*.** Head and mesosoma green blue with diffuse coppery luster; antenna with scape, pedicel and anelli yellowish brown, F1–F6 and clava brown. Fore and hind coxa dark green with diffuse coppery luster, mid coxae yellow, all femora, tibiae and tarsi yellow. Fore wing hyaline, venation yellowish brown. Metasoma dorsally dark brown with metallic blue violet luster; ovipositor sheaths brown.

***Sculpture*.** Head and mesosoma reticulate; clypeus reticulate-striate; scutellum strongly reticulate, but frenal area finely reticulate; propodeum reticulate, nucha alutaceous; petiole smooth; metasoma smooth and shiny.

***Head*.** In dorsal view 2.17–2.40 × as broad as long and 1.40–1.60 × as broad as mesoscutum; in frontal view 1.61–1.72 × as broad as high. POL 0.47–0.59 × as long as OOL. Eye height 1.11–1.15 × eye length and 1.54–1.76 × malar space. Distance between antennal toruli and lower margin of clypeus 3.14–3.35 × distance between antennal toruli and median ocellus. Antenna with scape 0.95–1.07 × as long as eye height and 1.05–1.18 × as long as eye length; pedicel 1.00–1.10 × as long as broad; combined length of pedicel and flagellum 0.88–1.00 × breadth of head; F1–F6 longer than broad, F1 2.00–2.20 × as long as broad and with two or three rows of sensilla; clava 2.80–3.14 × as long as broad, with small microsetose area on C3 and C4. Lower posterior corner of gena with large spine. Lower margin of clypeus concave bilaterally, in middle part distinctly emarginate.

***Mesosoma*.** 1.13–1.23 × as long as broad. Scutellum moderately arched, 0.83–0.92 × as long as broad, frenal area differentiated by a change in sculpture. Propodeum 0.63–0.74 × as long as scutellum, without costula but with median carina, nucha not small. Fore wing 2.08–2.23 × as long as its maximum width; basal cell with 1–3 setae, basal vein setose; speculum closed below; M 0.68–0.86 × as long as PM and 1.05–1.09 × as long as S, stigma large.

***Metasoma*.** 1.47–1.78 × as long as broad, 1.04–1.26 × as long as mesosoma, 0.65–0.74 × as long as mesosoma and head. Petiole as long as broad. Ovipositor sheaths projecting slightly beyond apex of metasoma.

**Male.** Unknown. Previously discovered males have been re-identified as females.

##### Biology.

Egg parasitoids of hemipterans *Erthesina* sp. and *Placosternum dama* (Fabricius, 1794) (Pentatomidae) (UCD Community 2025).

##### Distribution.

India, Myanmar, Sri Lanka (UCD Community 2025).

##### Comments.

*Acroclisoides indicus* Ferrière belongs to a group of species that have a hyaline fore wing. This species is similar to *A. sativus* Kumar & Khan and *A. luzonensis* Gahan; the differences between these species are given in the key.

Material from China was misidentified as *A. indicus* Ferrière. Here this material is regarded as *A. bicolor* Luo & Qin, 1991.

#### ﻿ Acroclisoides laticeps

Taxon classificationAnimaliaHymenopteraPteromalidae

Girault & Dodd, 1915

DE803DB0-2B19-569F-86BD-1388A43F9056

Figs 49–56

Acroclisoides laticeps Girault & Dodd, 1915: 335. Holotype female (QMBA, examined).

##### Type material examined.

***Holotype*** • female, Australia, “*Acroclisoides laticeps* Dodd ♀”, “TYPE”, “HOLOTYPE Hy. 2805 E.C.D.1984”, “Photographed specimen”, “TYPE Hy. 2805 A.A. Girault” (QMBA).

**Figures 49–56.**
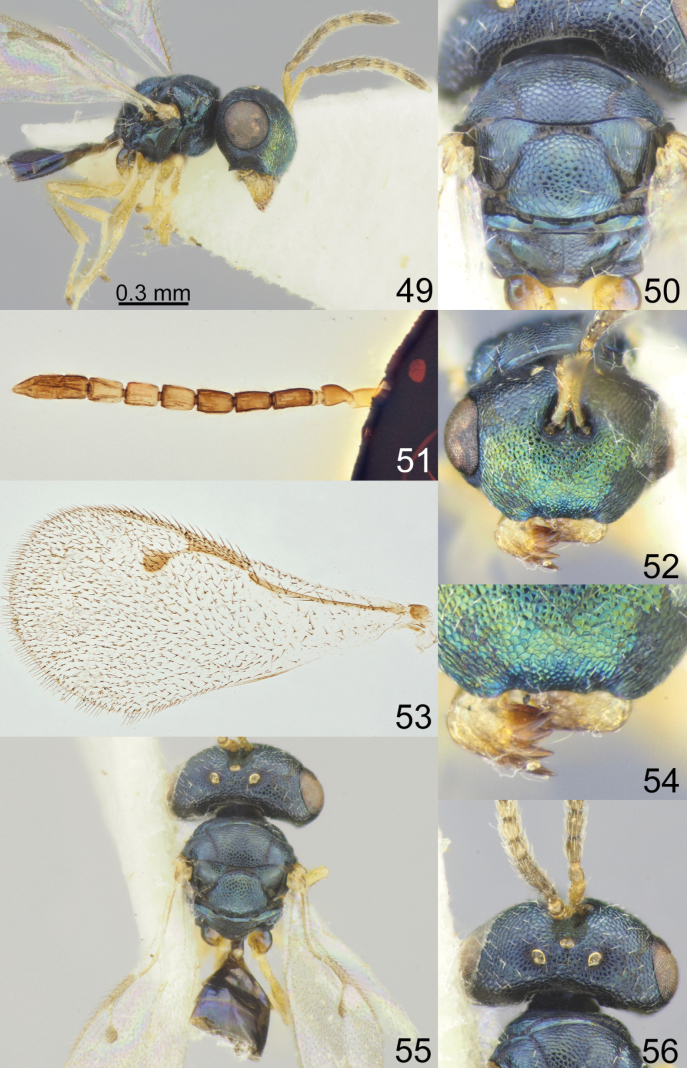
*Acroclisoides laticeps* Girault & Dodd, 1915, female, holotype 51, 53. and female, not type 49, 50, 52, 54–56. 49. Habitus, lateral view; 50. Mesosoma and propodeum, dorsal view; 51. Antenna; 52. Head, frontal view; 53. Fore wing; 54. Clypeus; 55. Habitus, dorsal view; 56. Head and pronotum, dorsal view.

##### Additional material examined.

Australia • 1 male, “S.E. QUEENSLAND Tambourine Mts. 11–17.V.1935”, “R.E. Turner. B.M. 1935-240”, “*Acroclisoides*”, “NHMUK 013455923” (NHMUK). Papua New Guinea • 4 females, “P.A.T.I. Popondetta N.D. 11.12.72 R. Berena”, “P. 1080”, “P. 1082”, “P. 1086”, “P. 1087”, “NHMUK 013455936”, “NHMUK 013455938”, “NHMUK 013455939”, “NHMUK 013455943” (NHMUK, ZISP) • 1 female, “PAPUA N. GUINEA Bulolo 13.XII.82 Bouček”, “*Acroclisoides*”, “NHMUK 013455905” (NHMUK).

##### Description.

**Female.** Body length 1.25–1.35 mm; fore wing length 1.20–1.25 mm.

***Coloration*.** Head and mesosoma dorsally dark blue with diffuse metallic luster; head frontally dark blue green with diffuse coppery luster; antenna with scape yellow, pedicel and anelli yellowish brown, F1–F3 and clava brown, F4–F6 yellow or yellowish brown. Fore and hind coxae dark brown with diffuse blue luster, mid coxae yellow, all femora, tibiae and tarsi yellow. Fore wing hyaline, venation brown. Metasoma dorsally dark brown with metallic blue violet luster; ovipositor sheaths brown.

***Sculpture*.** Head and mesosoma reticulate; clypeus reticulate; scutellum reticulate, frenal area finely reticulate; propodeum reticulate, nucha alutaceous; petiole smooth; metasoma smooth and shiny.

***Head*.** In dorsal view 2.04–2.25 × as broad as long and 1.34–1.41 × as broad as mesoscutum; in frontal view 1.47–1.55 × as broad as high. POL 0.74–0.75 × as long as OOL. Eye height 1.10–1.13 × eye length and 1.79–1.83 × malar space. Distance between antennal toruli and lower margin of clypeus 2.20–2.50 × distance between antennal toruli and median ocellus. Antenna with scape 0.85–0.88 × as long as eye height and 0.93–1.00 × as long as eye length; pedicel 1.25 × as long as broad; combined length of pedicel and flagellum 0.95–1.03 × breadth of head; F1–F6 longer than broad, F1 1.87–2.00 × as long as broad and with one or two rows of sensilla; clava 2.63–2.80 × as long as broad, with small microsetose area on C3 and C4. Lower posterior corner of gena forming an acute angle. Lower margin of clypeus concave bilaterally, in middle part emarginate.

***Mesosoma*.** 1.20 × as long as broad. Scutellum moderately arched, 1.23–1.26 × as long as broad, frenal area differentiated by a change in sculpture. Propodeum 0.58–0.65 × as long as scutellum, without costula but with median carina, nucha small. Fore wing 2.08–2.10 × as long as its maximum width; basal cell and basal vein setose; speculum closed below; M 0.75–0.82 × as long as PM and 1.00–1.07 × as long as S, stigma large.

***Metasoma*.** 1.25–1.30 × as long as broad, 0.83–0.88 × as long as mesosoma, 0.60–0.61 × as long as mesosoma and head. Petiole 1.00–1.11 × as long as broad. Ovipositor sheaths projecting slightly beyond apex of metasoma.

**Male.** Body length 1.20 mm; fore wing length 1.16 mm. Metasoma 1.48 × as long as broad. Otherwise, similar to female.

##### Biology.

Unknown.

##### Distribution.

Australia (Girault 1915), Papua New Guinea (new record).

##### Comments.

*Acroclisoides laticeps* Girault & Dodd belongs to a group of species that have a hyaline fore wing in both sexes. This species is similar to *A. suryai* Tselikh, sp. nov.; the differences between these species are given in the key.

#### ﻿ Acroclisoides luzonensis

Taxon classificationAnimaliaHymenopteraPteromalidae

Gahan, 1920

8B2ADF65-6369-57FE-8A59-B38263850EE5

Figs 57–64

Acroclisoides luzonensis Gahan, 1920: 345. Holotype female (USNM, not examined).

##### Material examined.

China • 3 females, “Huangfu Mt. 19?? VIII 26”, “Liao, Dingxi” (IZAS). Indonesia • 18 females, 4 males, “JAVA Buitenzorg 1937 J.S. Phillips Ex. *Chrysocoris atricapilla*”, “NHMUK 013455956”, “NHMUK 013455961”, “NHMUK 013455962”, “NHMUK 013455964”, “NHMUK 013455994”, “NHMUK 013455995”, “NHMUK 013455996”, “NHMUK 013455997”, “NHMUK 013456000”, “NHMUK 013456001”, “NHMUK 013456002”, “NHMUK 013456003”, “NHMUK 013456004”, “NHMUK 013456005”, “NHMUK 013456006”, “NHMUK 013456007”, “NHMUK 013456023” (NHMUK, ZISP) • 2 females, “W. Java. 1937 Ex *Chrysocoris atricapilla* J.S. Phillips”, “*Acroclisoides luzonensis* Gahan det. Subba Rao, 197”, “NHMUK 013455965” (NHMUK). Malaysia • 9 females, “MALAYA Kuala Lumpur Feb.12.1939”, “Ex F.M.S. Museum B.M. 1955-354”, “NHMUK 013455947”, “NHMUK 013455951”, “NHMUK 013455952” (NHMUK) • 2 males, “MALAYA Selangor Kuala Lumpur 7.VI.1954 H.T. Pagden 17780”, “Pres by Com Inst Ent B M 1957-248”, “COM. INST. ENT. COLL. NO.14126”, “Ex eggs of Menida varipennis”, “NHMUK 013455998”, “NHMUK 0134559998” (NHMUK) • 2 females “MALAYSIA: Selangor Kuala Lumpur; Univ. of Malaya, Rimba IIma 16.VI.90; J.M. Heraty; H093; sweep Eugenia”, “Univ. Calif. Riverside Ent. Res. Museum UCRC ENT 508657”, “*Acroclisoides* det. Xiao Hui 2004” (UCR). Republic of Korea • 1 female, “S. Korea: Gyeongsangnam-do (GN), Geochang-gun, SNME 35°44'54"N, 127°56'26"E, 22.VI.2022 coll. Tselikh” (NIBR).

**Figures 57–64.**
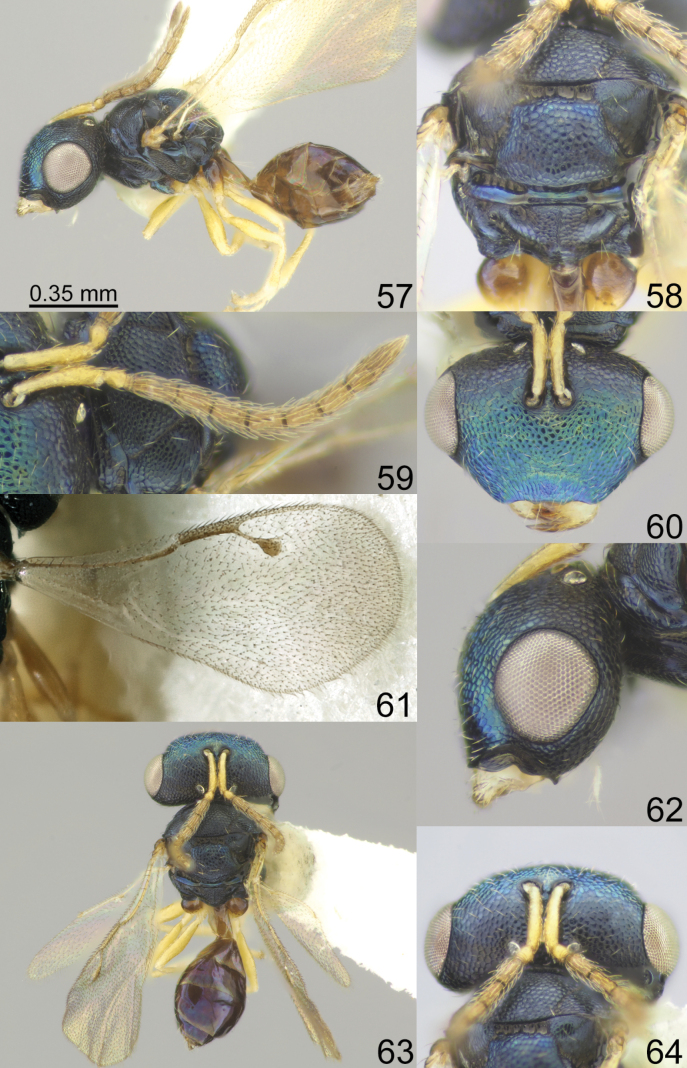
*Acroclisoides luzonensis* Gahan, 1920, female, not type. 57. Habitus, lateral view; 58. Mesosoma and propodeum, dorsal view; 59. Antenna; 60. Head, frontal view; 61. Fore wing; 62. Head, lateral view; 63. Habitus, dorsal view; 64. Head and pronotum, dorsal view.

##### Description.

**Female.** Body length 1.40–2.00 mm; fore wing length 1.35–1.60 mm.

***Coloration*.** Head and mesosoma dorsally dark blue with diffuse metallic luster; head frontally dark blue with diffuse green coppery luster; antenna with scape and pedicel yellow, anelli yellowish brown, F1–F6 and clava brown. Fore and hind coxa dark blue with metallic diffuse luster, mid coxae yellowish brown, all femora, tibiae and tarsi yellow. Fore wing hyaline, venation brown. Metasoma dorsally dark brown with metallic blue violet luster; ovipositor sheaths brown.

***Sculpture*.** Head and mesosoma reticulate; clypeus reticulate-striate; scutellum strongly reticulate, but frenal area finely reticulate; propodeum reticulate, nucha alutaceous; petiole smooth; metasoma smooth and shiny.

***Head*.** In dorsal view 2.38–2.30 × as broad as long and 1.39–1.45 × as broad as mesoscutum; in frontal view 1.61–1.70 × as broad as high. POL 0.68–0.70 × as long as OOL. Eye height 1.13–1.14 × eye length and 2.35–2.61 × malar space. Distance between antennal toruli and lower margin of clypeus 2.43–2.50 × distance between antennal toruli and median ocellus. Antenna with scape 1.00–1.06 × as long as eye height and 1.13–1.20 × as long as eye length; pedicel 1.00–1.25 × as long as broad; combined length of pedicel and flagellum 0.93–0.94 × breadth of head; F1–F6 longer than broad, F1 2.10–2.20 × as long as broad and with two rows of sensilla; clava 2.22–2.33 × as long as broad, with small microsetose area on C3 and C4. Lower posterior corner of gena with small sharp spine. Lower margin of clypeus concave bilaterally, in middle part shallowly emarginate.

***Mesosoma*.** 1.16–1.21 × as long as broad. Scutellum moderately arched, 0.83 × as long as broad, frenal area differentiated by a change in sculpture. Propodeum 0.63–0.80 × as long as scutellum, without costula but with median carina, nucha small. Fore wing 2.03–2.17 × as long as its maximum width; basal cell partly setose, basal vein setose; speculum closed below; M 0.85–0.86 × as long as PM and 1.16–1.30 × as long as S, stigma large.

***Metasoma*.** 1.78–2.40 × as long as broad, 0.97–1.15 × as long as mesosoma, 0.69–0.78 × as long as mesosoma and head. Petiole 1.25 × as long as broad. Ovipositor sheaths projecting slightly beyond apex of metasoma.

**Male.** Body length 1.53–1.80 mm; fore wing length 1.38–1.67 mm. Eye height 1.93–2.00 × malar space. Distance between antennal toruli and lower margin of clypeus 2.00–2.10 × distance between antennal toruli and median ocellus. Metasoma 2.58–2.60 × as long as broad. Otherwise, similar to female.

##### Biology.

Egg parasitoids of hemipterans *Eucorysses javanus* (Westwood, 1837) (new host record) and *Tectocoris lineola* (Fabricius, 1781) (Scutelleridae) and some Pentatomidae species. Hyperparasitoids of *Trissolcus banksi* (Gahan, 1921) (Scelionidae) (UCD Community, 2025).

##### Distribution.

China (UCD Community 2025), Indonesia (new record), Malaysia (new record), Philippines (UCD Community 2025), Republic of Korea (new record).

##### Comments.

*Acroclisoides luzonensis* Gahan belongs to a group of species that have a hyaline fore wing in both sexes. This species is similar to *A. major* Girault & Dodd and *A. nongae* Tselikh, Lee & Ku, sp. nov.; the differences between these species are given in the key.

#### ﻿ Acroclisoides maculatus

Taxon classificationAnimaliaHymenopteraPteromalidae

Sureshan & Narendran, 2002

2576879A-97A1-5231-8C18-DA62417A45F7

Figs 65–72

Acroclisoides maculatus Sureshan & Narendran, 2002: 127,128–130. Holotype female (ZSI, not examined).

##### Material examined.

India • 4 females, “INDIA. T.N. Padappai 24.I.86 Alexander”, “Sp. No. 13 CIE 18043”, “*Acroclisoides indicus* Ferr. det. Z. Bouček, 1986”, “NHMUK 013455882”, “NHMUK 013455968”, “NHMUK 013458845”, “NHMUK 013458847” (NHMUK) • 3 females, “INDIA: Madras 1976, no. 17 Anantakrishnan”, “NHMUK 013455967”, “NHMUK 013455969” (NHMUK) • 2 females, “INDIA: Madras 1976, no. 3 Anantakrishnan”, “*Acroclisoides indicus* Ferr. det. Z. Bouček, 1977”, “NHMUK 013458846” (NHMUK) • 3 females, “INDIA: Madras, Pentatomid eggs 1977. Thirumalai”, “*A. indicus*”, “NHMUK 013455973” (NHMUK).

**Figures 65–72.**
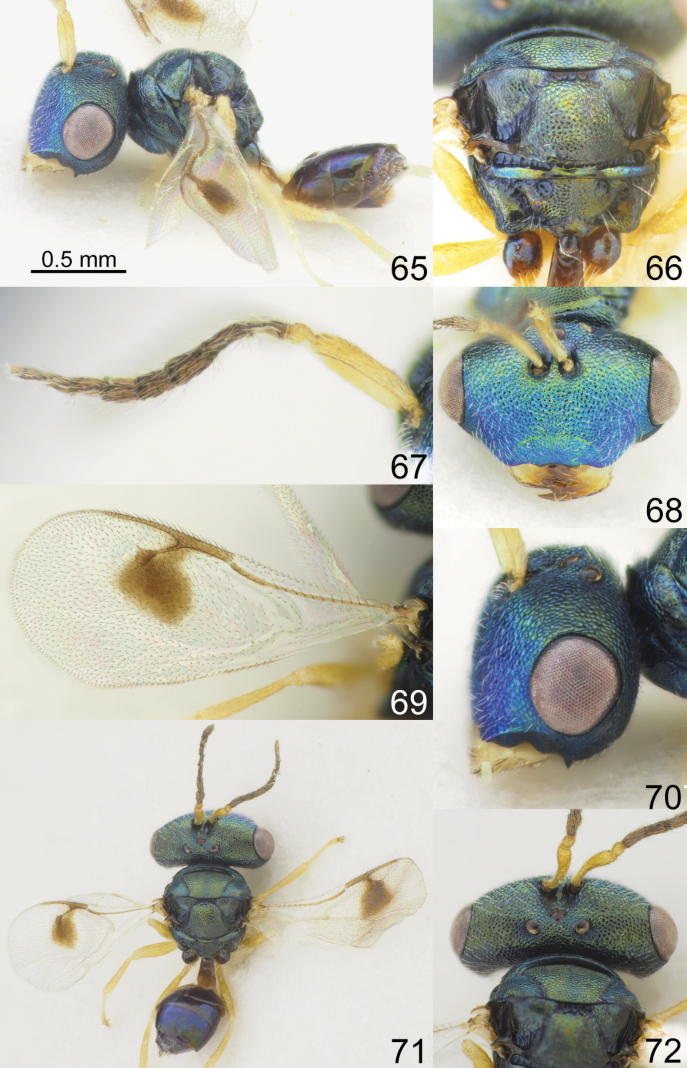
*Acroclisoides maculatus* Sureshan & Narendran, 2002, female, not type. 65. Habitus, lateral view; 66. Mesosoma and propodeum, dorsal view; 67. Antenna; 68. Head, frontal view; 69. Fore wing; 70. Head, lateral view; 71. Habitus, dorsal view; 72. Head and pronotum, dorsal view.

##### Description.

**Female.** Body length 1.70–2.10 mm; fore wing length 1.60–1.70 mm.

***Coloration*.** Head and mesosoma dorsally dark green with diffuse metallic diffuse coppery luster; head frontally dark blue green with diffuse metallic coppery luster; antenna with scape, pedicel and anelli yellow, F1–F6 and clava brown. Fore and hind coxa dark blue with diffuse metallic luster, mid coxae yellowish brown, all femora, tibiae and tarsi yellow. Fore wing with one spot near S, venation brown. Metasoma dorsally dark brown with metallic blue violet luster; ovipositor sheaths brown.

***Sculpture*.** Head, clypeus and mesosoma reticulate; scutellum, frenal area and propodeum strongly reticulate, nucha alutaceous; petiole smooth; metasoma smooth and shiny.

***Head*.** In dorsal view 2.23–2.43 × as broad as long and 1.35–1.46 × as broad as mesoscutum; in frontal view 1.57–1.67 × as broad as high. POL 0.54–0.60 × as long as OOL. Eye height 1.12–1.15 × eye length and 1.50–1.63 × malar space. Distance between antennal toruli and lower margin of clypeus 3.35–4.60 × distance between antennal toruli and median ocellus. Antenna with scape 0.94–1.03 × as long as eye height and 1.06–1.19 × as long as eye length; pedicel 1.12–1.45 × as long as broad; combined length of pedicel and flagellum 0.72–0.83 × breadth of head; F1–F6 longer than broad, F1 1.64–1.83 × as long as broad and with two rows of sensilla; clava 2.46–2.67 × as long as broad, with small microsetose area on C3 and C4. Lower posterior corner of gena with small sharp spine. Lower margin of clypeus concave bilaterally, in middle part distinctly emarginate.

***Mesosoma*.** 1.14–1.16 × as long as broad. Scutellum moderately arched, 0.83 × as long as broad, frenal area differentiated by a change in sculpture. Propodeum 0.72–0.75 × as long as scutellum, without costula but with median carina, nucha small. Fore wing 2.20–2.24 × as long as its maximum width; basal cell with 0–3 setae, basal vein setose; speculum closed below; M 0.67–0.73 × as long as PM and 1.17–1.22 × as long as S, stigma large.

***Metasoma*.** 1.60–1.80 × as long as broad, 0.70–1.12 × as long as mesosoma, 0.53–0.80 × as long as mesosoma and head. Petiole 1.00–1.50 × as long as broad. Ovipositor sheaths projecting slightly beyond apex of metasoma.

**Male.** Body length 1.70 mm; fore wing length 1.58 mm. Antenna thicker than in female; gaster short and compressed. Otherwise, similar to female (Sureshan and Narendran 2002).

##### Biology.

Egg parasitoids of hemipterans of the family Pentatomidae (Sureshan and Narendran 2002).

##### Distribution.

India (Sureshan and Narendran 2002).

##### Comments.

*Acroclisoides maculatus* Sureshan & Narendran belongs to a group of species where females have a fore wing with one spot. This species is similar to *A. megacephalus* Girault & Dodd; the differences between these species are given in the key.

The material from India was misidentified as *A. indicus* Ferrière by Bouček (see Material examined above). Here this material is regarded as *A. maculatus* Sureshan & Narendran.

#### ﻿ Acroclisoides major

Taxon classificationAnimaliaHymenopteraPteromalidae

Girault & Dodd, 1915

ECDCBC1C-1FAC-5FEC-9DA8-50E57309E4DE

Figs 73–80

Acroclisoides major Girault & Dodd, 1915: 335. Holotype female (QMBA, examined).

##### Type material examined.

***Holotype*** • female, Australia, “*Acroclisoides major* Dodd ♀”, “TYPE”, “HOLOTYPE Hy. 2806 E.C.D.1984”, “Photographed specimen” (QMBA).

**Figures 73–80.**
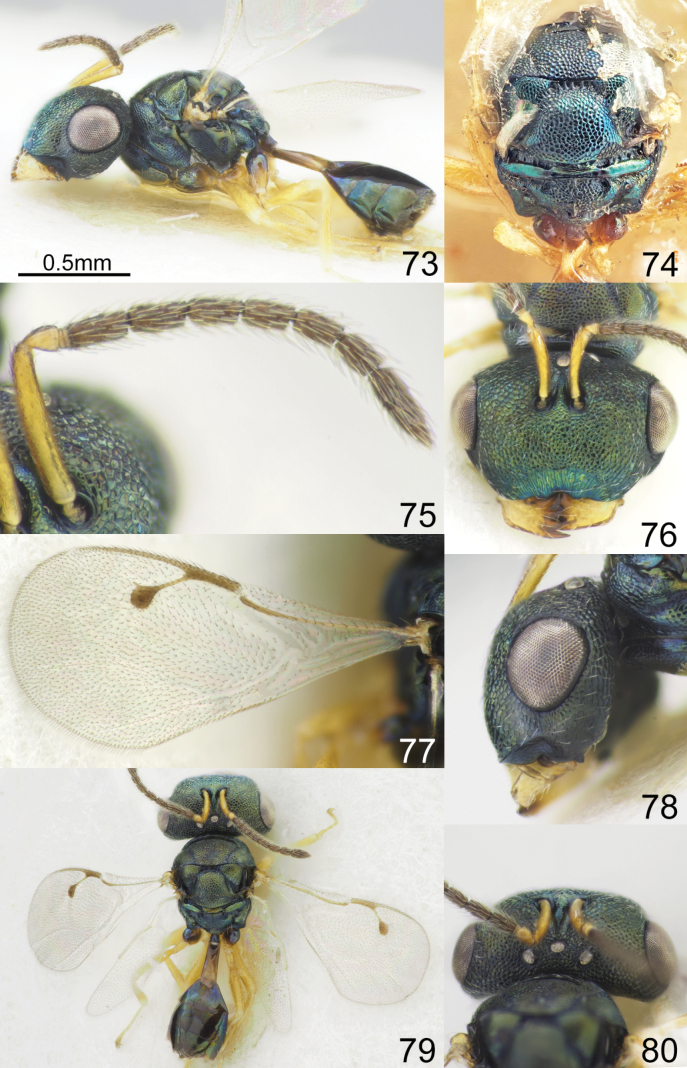
*Acroclisoides major* Girault & Dodd, 1915, female, holotype 74. and female, not type 73, 75–80. 73. Habitus, lateral view; 74. Mesosoma and propodeum, dorsal view; 75. Antenna; 76. Head, frontal view; 77. Fore wing; 78. Head, lateral view; 79. Habitus, dorsal view; 80. Head and pronotum, dorsal view.

##### Additional material examined.

Australia • 1 female, “Exp. F.W. 131 Date. 30.IV.53 Frank Wilson. AUSTRALIA”, “COM. INST. ENT. COLL. NO 13221”, “Pres by Com Inst Ent B M 1953-623”, “NHMUK 013455920” (NHMUK) • 1 female, “Exp. F.W. 131 Date. 1.V.53 Frank Wilson. AUSTRALIA”, “COM. INST. ENT. COLL. NO 13221”, “Pres by Com Inst Ent B M 1953-623”, “*Acroclisoides* sp. ♂ G.J. Kerrich det. 1953”, “♀”, “NHMUK 013455914” (NHMUK) • 2 females, “Exp. F.W. 214.2 Date. 27.I.54 Frank Wilson. AUSTRALIA”, “COM. INST. ENT. COLL. NO 13611”, “AUSTRALIA”, *Acroclisoides* sp. G.J. Kerrich det. 1954”, “NHMUK 013455945” (NHMUK) • 1 female, “AUSTRALIA: N.S.W. Canley Vale 28.V.1961 M. Nikitin B. M. 1961-717.”, “NHMUK 013455940” (NHMUK) • 2 females, “AUSTRALIA: N.S.W. Cabramatta 2.X.1961”, “M. Nikitin B. M. 1962-101”, “*Acroclisoides*”, “NHMUK 013455942” (NHMUK) • 1 female, “AUSTRALIA: N.S.W. Caramar 15.X.1961 M. Nikitin B. M. 1962-101”, “NHMUK 013455932” (NHMUK) • 1 female, “AUSTRALIA: N.S.W. Nr. Liverpool, Hoxton Park. 8.III.1965”, “NHMUK 013455883” (NHMUK) • 1 female, “QUEENSLAND: 15 km SE. of Nambour 6.XI.76 Z. Bouček”, “NHMUK 013455934” (NHMUK) • 3 females, “Brisbane-Indooroopilly, QNSLD. XII.76 Z. Bouček”, “*Acroclisoides* ♀”, “NHMUK 013455916”, “NHMUK 013455941”, “NHMUK 013455944” (NHMUK) • 2 females, “SE. QUEENSLAND: Bribie Island 22.XII.76 Z. Bouček”, “NHMUK 013455929” (ZISP) • 1 female, “Melbourne, VICT. 31.I.77 Z. Bouček”, “*Acroclisoides*”, “NHMUK 013455908” (NHMUK) • 1 female, “N. N.S. WALES: Tooloom Scruo 8.I.77 Z. Bouček”, “*Acroclisoides* ♀”, “NHMUK 013455924” (NHMUK) • 1 female, “Samford nr. Brisbane, QUEENSLAND 16.I.77 Z. Bouček”, “NHMUK 013455931” (NHMUK) • 1 female, “SE. QUEENSLAND Mt. Tamborine”, “Malaise Trap XI.77”, “NHMUK 013455935” (NHMUK) • 4 females, “5 km south of Mylor, S. Aust. 29.I.79”, “A.D. Austin.”, “Eggs Heteroptera collect under bank Eucalyptus viminalis”, “NHMUK 013455948”, “NHMUK 013455949”, “NHMUK 013455950”, “NHMUK 013455953” (NHMUK) • 1 female, “Qld., AUSTRALIA Cooloola St. For. Camp Milo 30-x-79 EC Dahms & LaSalle”, “Univ. Calif. Riverside Ent. Res. Museum UCRC ENT 508660” (UCR) • 1 female, “Qld., AUSTRALIA 19 km W Gordonvale Goldsborough Rd. malaise, rain forest 12–22-xi-79”, “Univ. Calif. Riverside Ent. Res. Museum UCRC ENT 508659” (UCR) • 1 male, “SEQld., AUSTRALIA Gatton 4-xii-79 Coll. E.C. Dahms”, “Univ. Calif. Riverside Ent. Res. Museum UCRC ENT 508662” (UCR) • 2 females, “Canberra. 9.I.80 A.D. Austin”, “Australia ACT”, “NHMUK 013455958”, “NHMUK 013455959” (NHMUK) • 2 females, “AUSTRALIA, A.C.T. Canberra (Bl.Mtn.) M.t. II.81 J. Short”, “*Acroclisoides* ♀ det. Bouček, 1985”, “NHMUK 013455930”, “NHMUK 013455937” (NHMUK) • 1 female, “AUSTRALIA; Old. Ipswich dist. 3.vi.1980”, “J.S. Noyes B.M. 1981-299”, “*Acroclisoides*”, “NHMUK 013455917” (NHMUK) • 1 male, “N. QUEENSLAND Atherton distr. 5.xii.82 Bouček”, “*Acroclisoides* ♂ det. Bouček, 1984”, “NHMUK 013455909” (NHMUK) • 1 male, “AUS, Tasmania; Mt. Field N.P. 10/2.84 Masner”, “NHMUK 013455982” (ZISP).

##### Description.

**Female.** Body length 1.95–2.10 mm; fore wing length 1.80–1.90 mm.

***Coloration*.** Head and mesosoma dorsally dark blue with diffuse metallic diffuse luster; head frontally dark green with diffuse metallic coppery luster; antenna with scape, pedicel and anelli yellowish brown, F1–F6 and clava brown. Fore and hind coxa dark blue with metallic green and diffuse violet luster, mid coxae yellowish brown, all femora, tibiae and tarsi yellow. Fore wing hyaline, venation brown. Metasoma dorsally dark brown with metallic blue, green and violet luster; ovipositor sheaths brown.

***Sculpture*.** Head, clypeus and mesosoma reticulate; scutellum, frenal area and propodeum strongly reticulate, nucha finely reticulate; petiole smooth; metasoma smooth and shiny.

***Head*.** In dorsal view 2.11–2.38 × as broad as long and 1.32–1.37 × as broad as mesoscutum; in frontal view 1.47–1.49 × as broad as high. POL 0.90–0.92 × as long as OOL. Eye height 1.13–1.17 × eye length and 1.79–1.81 × malar space. Distance between antennal toruli and lower margin of clypeus 1.92–2.22 × distance between antennal toruli and median ocellus. Antenna with scape 0.90–0.94 × as long as eye height and 1.05–1.07 × as long as eye length; pedicel 1.40–1.55 × as long as broad; combined length of pedicel and flagellum 0.90–0.92 × breadth of head; F1–F6 longer than broad, F1 1.90–2.20 × as long as broad and with two or three rows of sensilla; clava 2.54–2.72 × as long as broad, with small microsetose area on C3 and C4. Lower posterior corner of gena with small sharp spine. Lower margin of clypeus concave bilaterally, in middle part deeply emarginate.

***Mesosoma*.** 1.24–1.35 × as long as broad. Scutellum moderately arched, 0.75–0.80 × as long as broad, frenal area differentiated by a change in sculpture. Propodeum 0.81–0.89 × as long as scutellum, without costula but with irregular median carina, nucha small. Fore wing 2.03–2.09 × as long as its maximum width; basal cell partly setose, basal vein setose; speculum closed below; M 0.65–0.81 × as long as PM and 0.85–1.00 × as long as S, stigma large.

***Metasoma*.** 1.57–1.63 × as long as broad, 0.89–1.04 × as long as mesosoma, 0.71–0.79 × as long as mesosoma and head. Petiole 1.00–1.25 × as long as broad. Ovipositor sheaths projecting slightly beyond apex of metasoma.

**Male.** Hitherto unknown. Body length 1.40–1.50 mm; fore wing length 1.25–1.35 mm. Metasoma 2.20–2.23 × as long as broad. Otherwise, similar to female.

##### Biology.

Unknown.

##### Distribution.

Australia (Girault 1915).

##### Comments.

*Acroclisoides major* Girault & Dodd belongs to a group of species that have a hyaline fore wing in both sexes. This species is similar to *A. nongae* Tselikh, Lee & Ku, sp. nov.; the differences between these species are given in the key.

#### ﻿ Acroclisoides marimbae

Taxon classificationAnimaliaHymenopteraPteromalidae

Tselikh & Mitroiu sp. nov.

3A4B1A1A-EC37-5BFB-99DD-894B78D0BD70

https://zoobank.org/2F1E5844-27F8-4AA5-873F-E289D71D51FD

Figs 81–88

##### Type material.

***Holotype*** • female, Tanzania, “TANGANYIKA Dar-es-Salaam ex Pentatomid 14.III.1919 W.A. Lambourn”, “Acroclisoides near luzonensis Gah. G.J. Kerrich det. 1948”, “NHMUK 013455889” (NHMUK). ***Paratypes*** • 7 females, 2 males, Senegal, “Senegal, Bambey, 12.VIII.1943, J. Risbec, Ex eggs of Pentatomia, 486” (1 male bearing an additional label: “Pachyneuronini ? genus, G. Nixon det. 1947”) (NHMUK) • 4 females, 4 males, Senegal, “Senegal, Bambey, J. Risbec, 484, 533”, “Ex Atalecore notatipennis” (NHMUK) • 3 females, Zimbabwe, “Rhodesia: Salisbury, v.1979, A. Watsham” (NHMUK, MICO) • 1 female, Zimbabwe, “Zimbabwe: Harar, St. Ignatius, V.1990, A. Watsham”, “Acroclisoides det. R. Burks 2004”, “CNC L-2017-073” (CNC) • 2 females, 1 male, Tanzania: “TANGANYIKA T. Dar es Salaam W.A. Lambourn Ex ova on leaf. Casuarina Em. 20.III.1919”, “429”, “NHMUK 013455888”, “NHMUK 013455890”, “NHMUK 013455891” (NHMUK, ZISP).

**Figures 81–88.**
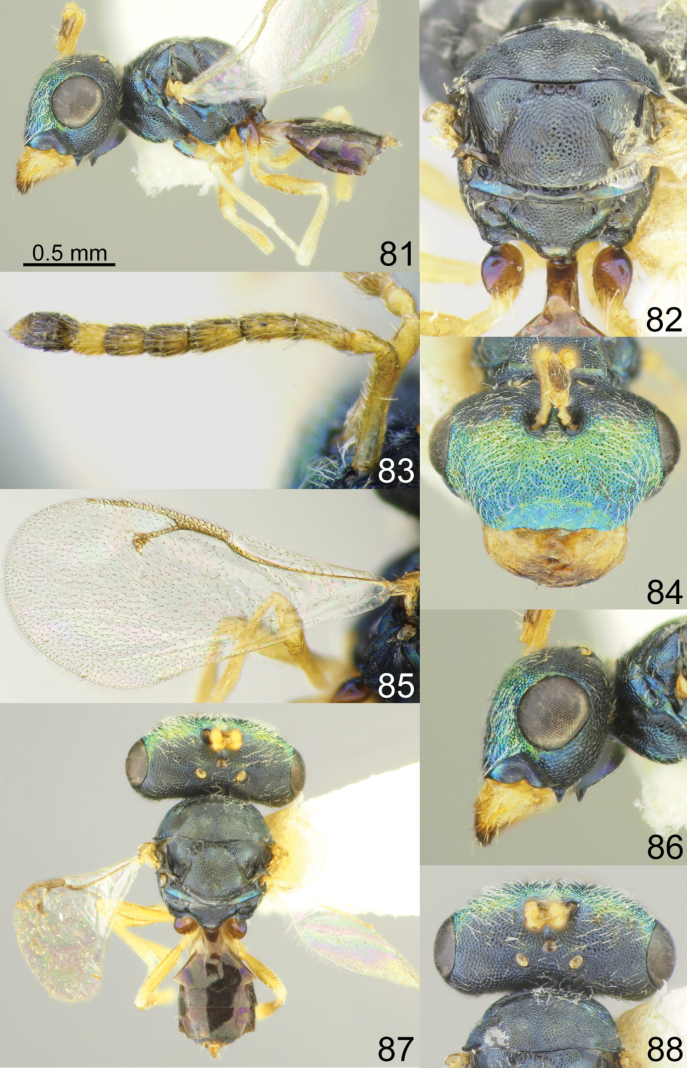
*Acroclisoides marimbae* Tselikh & Mitroiu, sp. nov., female, holotype. 81. Habitus, lateral view; 82. Mesosoma and propodeum, dorsal view; 83. Antenna; 84. Head, frontal view; 85. Fore wing; 86. Head, lateral view; 87. Habitus, dorsal view; 88. Head and pronotum, dorsal view.

##### Description.

**Female.** Body length 1.90–2.00 mm; fore wing length 1.70–1.75 mm.

***Coloration*.** Head and mesosoma dorsally black with diffuse metallic blue luster; head frontally dark blue green with diffuse metallic coppery luster; antenna with scape, pedicel, anelli and F1–F3 yellowish brown, F4–F5 brown, F6 yellow, clava with C1–C2 brown and C3–C4 yellowish brown. Fore and hind coxae dark blue green with diffuse metallic coppery and violet luster, mid coxae yellowish brown, all femora yellowish brown, all tibiae and tarsi yellow. Fore wing hyaline, venation brown. Metasoma dorsally dark brown with metallic violet luster; ovipositor sheaths brown.

***Sculpture*.** Head, clypeus and mesosoma reticulate; scutellum strongly reticulate, frenal area finely reticulate, propodeum finely reticulate, nucha alutaceous; petiole smooth; metasoma smooth and shiny.

***Head*.** In dorsal view 2.29–2.35 × as broad as long and 1.55–1.62 × as broad as mesoscutum; in frontal view 1.55–1.71 × as broad as high. POL 0.63–0.71 × as long as OOL. Eye height 1.06–1.12 × eye length and 1.40–1.46 × malar space. Distance between antennal toruli and lower margin of clypeus 4.50–5.10 × distance between antennal toruli and median ocellus. Antenna with scape 1.05–1.08 × as long as eye height and 1.18–1.21 × as long as eye length; pedicel 1.00–1.18 × as long as broad; combined length of pedicel and flagellum 0.55–0.87 × breadth of head; F1–F6 longer than broad, F1 1.83–2.20 × as long as broad and with two or three rows of sensilla; clava 2.20–2.40 × as long as broad, with small microsetose area on C3 and C4. Lower posterior corner of gena with large, sharp spine. Lower margin of clypeus concave bilaterally, in middle part broadly emarginate.

***Mesosoma*.** 1.27–1.34 × as long as broad. Scutellum moderately arched, 0.88–0.91 × as long as broad, frenal area differentiated by sculpture. Propodeum 0.78–0.80 × as long as scutellum, without costula or median carina, nucha small. Fore wing 2.08–2.14 × as long as its maximum width; basal cell with 5–7 setae, basal vein setose; speculum closed below; M 0.60–0.74 × as long as PM and 1.00–1.22 × as long as S, stigma small to moderate.

***Metasoma*.** 1.52–1.55 × as long as broad, 0.79–0.93 × as long as mesosoma, 0.56–0.72 × as long as mesosoma and head. Petiole 1.00–1.10 × as long as broad. Ovipositor sheaths projecting slightly beyond apex of metasoma.

**Male.** Body length 1.80 mm. Head posterior to malar space striate reticulate. Distance between antennal toruli and lower margin of clypeus 3.45 × distance between antennal toruli and median ocellus. Metasoma 1.65 × as long as broad. Antenna with F1–F6 yellowish brown. Otherwise, similar to female.

##### Etymology.

The species is named in honor of the queen Marimba, a folk hero whose accomplishments have become part of the African folklore (noun in genitive case).

##### Biology.

The species was reared from eggs of *Atelocera* (as *Atalecore*) *notatipennis* Stål, 1858 (Hemiptera: Pentatomidae) (see above), but it is not clear if they are primary parasitoids of hyperparasitoids.

##### Distribution.

Kenya, Senegal, Tanzania, Zimbabwe.

##### Comments.

*Acroclisoides marimbae* Tselikh & Mitroiu, sp. nov. belongs to a group of species that have a hyaline fore wing in both sexes. This species is similar to *A. bicolor* Luo & Qin; the differences between these species are given in the key.

#### ﻿ Acroclisoides megacephalus

Taxon classificationAnimaliaHymenopteraPteromalidae

Girault & Dodd, 1915

4ED83A63-F432-5456-BFC2-0986064D04C5

Figs 89–96

Acroclisoides megacephalus Girault & Dodd, 1915: 334. Holotype female (QMBA, examined).

##### Type material.

***Holotype*** • female, Australia, “*Acroclisoides megacephalus* Girault & Dodd ♀”, “TYPE”, “HOLOTYPE Hy. 2804 E.C.D.1984”, “Photographed specimen” (QMBA).

**Figures 89–96.**
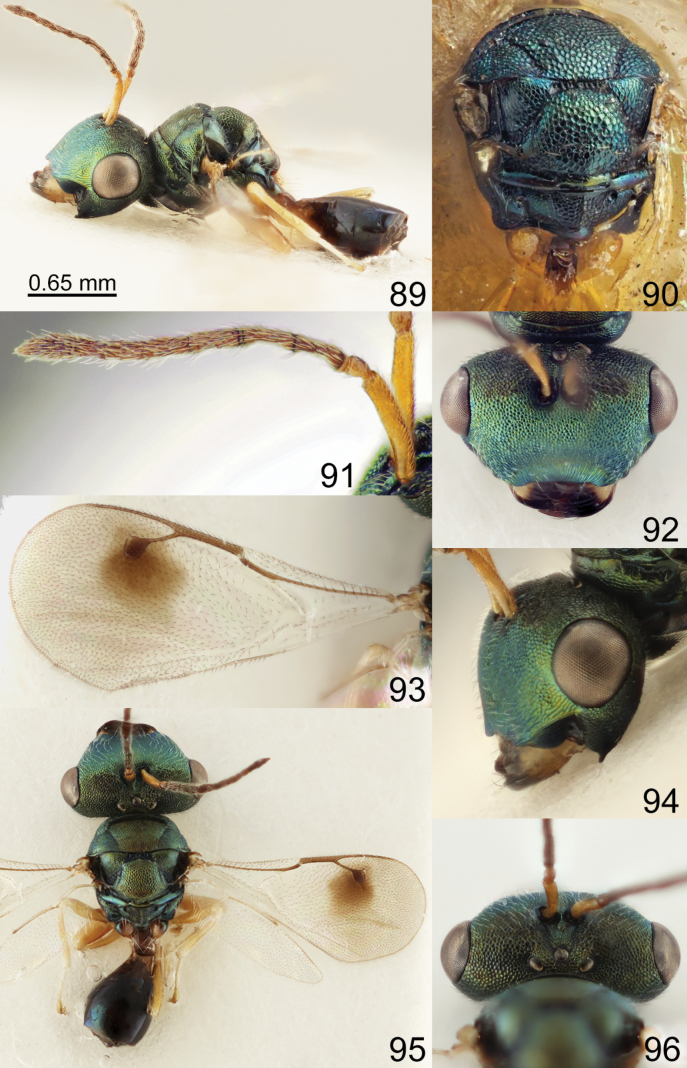
*Acroclisoides megacephalus* Girault & Dodd, 1915, female, holotype 90. and female, not type 89, 91–96. 89. Habitus, lateral view; 90. Mesosoma and propodeum, dorsal view; 91. Antenna; 92. Head, frontal view; 93. Fore wing; 94. Head, lateral view; 95. Habitus, dorsal view; 96. Head and pronotum, dorsal view.

##### Additional material examined.

Australia • 1 female, “Cooloola N:P. 7.III.84 L. Masner S. QUEENSLAND”, “*Acroclisoides* ♀ det. Z. Bouček, 1984”, “NHMUK 013455983” (ZISP) • 1 female, Australia, “SE. QUEENSLAND Mt. Tamborine XI.1977 Mal. Trap.”, “*Acroclisoides megacephalus* Gir. det. Z. Bouček, 1985”, “NHMUK 013455913” (NHMUK).

##### Description.

**Female.** Body length 2.60–2.70 mm; fore wing length 2.00–2.10 mm.

***Coloration*.** Head and mesosoma dorsally blue green with diffuse metallic coppery luster; antenna with scape, pedicel, anelli yellowish brown, F1–F6 and clava brown. Fore and hind coxae dark blue green with diffuse metallic coppery luster, mid coxae yellowish brown, all femora yellowish brown, all tibiae and tarsi yellow. Fore wing with one spot near S, venation brown. Metasoma dorsally dark brown with metallic violet and blue luster; ovipositor sheaths brown.

***Sculpture*.** Head and mesosoma reticulate; clypeus striate; scutellum, frenal area and propodeum strongly reticulate, nucha alutaceous; petiole smooth; metasoma smooth and shiny.

***Head*.** In dorsal view 2.54–2.59 × as broad as long and 1.40–1.42 × as broad as mesoscutum; in frontal view 1.64–1.65 × as broad as high. POL 0.55–0.57 × as long as OOL. Eye height 1.10–1.13 × eye length and 1.65–1.70 × malar space. Distance between antennal toruli and lower margin of clypeus 3.07–3.40 × distance between antennal toruli and median ocellus. Antenna with scape 0.94–0.96 × as long as eye height and 1.05–1.07 × as long as eye length; pedicel 1.20–1.33 × as long as broad; combined length of pedicel and flagellum 0.88–0.95 × breadth of head; F1–F6 longer than broad, F1 2.18–2.20 × as long as broad and with three rows of sensilla; clava 2.72–3.00 × as long as broad, with small microsetose area on C3 and C4. Lower posterior corner of gena with sharp spine. Lower margin of clypeus concave bilaterally, in middle part weakly emarginate, near straight.

***Mesosoma*.** 1.15–1.18 × as long as broad. Scutellum moderately arched, 0.77–0.85 × as long as broad, frenal area distinguished by a change in sculpture. Propodeum 0.70–0.72 × as long as scutellum, without costula but with median carina, nucha small. Fore wing 2.21–2.25 × as long as its maximum width; basal cell with 6–7 setae, basal vein setose; speculum closed below; M 0.73–0.85 × as long as PM and 1.18–1.22 × as long as S, stigma large.

***Metasoma*.** 1.81–1.90 × as long as broad, 1.04–1.25 × as long as mesosoma, 0.68–0.85 × as long as mesosoma and head. Petiole 0.50–0.60 × as long as broad. Ovipositor sheaths projecting slightly beyond apex of metasoma.

**Male.** Unknown.

##### Biology.

Egg parasitoids of the hemipteran *Axiagastus cambelli* Distant (Pentatomidae) (UCD Community, 2025).

##### Distribution.

Australia (Girault 1915).

##### Comments.

*Acroclisoides megacephalus* Girault & Dodd belongs to a group of species where females have a fore wing with one spot near S. This species is similar to *A. maculatus* Sureshan & Narendran; the differences between these species are given in the key.

#### ﻿ Acroclisoides miklukhai

Taxon classificationAnimaliaHymenopteraPteromalidae

Tselikh sp. nov.

E475C35B-79E5-5213-A8B8-4E79A453F554

https://zoobank.org/0740E4C2-5AF3-4F25-9693-E2F7BE813A57

Figs 97–104

##### Type material.

***Holotype*** • female, Papua New Guinea, “NEW GUINEA East New Britain 30.XII.1969”, “Ex. Eggs of *Axiagastus* sp. C.I.E. A 3688”, “*Acroclisoides megacephalus* Gir. B.R. Subba Rao det. 70”, “NHMUK 013455919” (NHMUK). ***Paratype*** • 1 female, Papua New Guinea, same data as holotype (ZISP).

**Figures 97–104.**
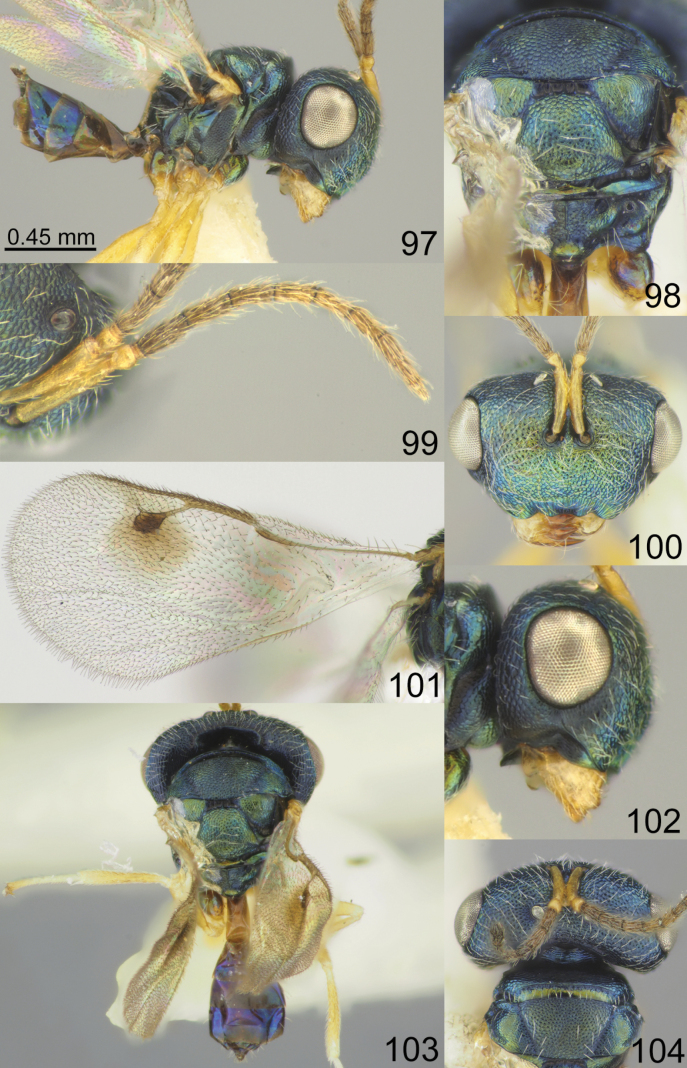
*Acroclisoides miklukhai* Tselikh, sp. nov., female, holotype. 97. Habitus, lateral view; 98. Mesosoma and propodeum, dorsal view; 99. Antenna; 100. Head, frontal view; 101. Fore wing; 102. Head, lateral view; 103. Habitus, dorsal view; 104. Head and pronotum, dorsal view.

##### Description.

**Female.** Body length 1.70–1.80 mm; fore wing length 1.50–1.60 mm.

***Coloration*.** Head, pronotum, mesoscutum and propodeum dorsally dark blue with diffuse metallic coppery luster; head frontally, axilla and scutellum dorsally green blue with diffuse metallic coppery luster; antenna with scape, pedicel and anelli yellowish brown, F1–F6 and clava brown. Fore and hind coxa dark blue green with diffuse metallic coppery luster, mid coxae yellowish brown, all femora yellowish brown, all tibiae and tarsi yellow. Fore wing with one spot near S, venation brown. Metasoma dorsally dark brown with metallic violet and blue luster; ovipositor sheaths brown.

***Sculpture*.** Head and mesosoma reticulate; clypeus striate; scutellum, frenal area and propodeum reticulate, nucha alutaceous; petiole smooth; metasoma smooth and shiny.

***Head*.** Head in dorsal view 2.07–2.20 × as broad as long and 1.32–1.41 × as broad as mesoscutum; in frontal view 1.59–1.63 × as broad as high. POL 0.62–0.68 × as long as OOL. Eye height 1.09–1.13 × eye length and 1.89–2.00 × malar space. Distance between antennal toruli and lower margin of clypeus 1.77–1.78 × distance between antennal toruli and median ocellus. Antenna with scape 0.88–0.89 × as long as eye height and 0.96–1.00 × as long as eye length; pedicel 1.00–1.20 × as long as broad; combined length of pedicel and flagellum 0.93–0.99 × breadth of head; F1–F6 longer than broad, F1 2.50–2.80 × as long as broad and with 2–3 rows of sensilla; clava 3.00–3.18 × as long as broad, with small microsetose area on C3 and C4. Lower posterior corner of gena forming an acute angle. Lower margin of clypeus concave bilaterally, in middle part distinctly emarginate.

***Mesosoma*.** Mesosoma 1.13–1.21 × as long as broad. Scutellum moderately arched, 0.76–0.87 × as long as broad, frenal area differentiated by a change in sculpture. Propodeum 0.72–0.75 × as long as scutellum, without costula but with median carina, nucha not small. Fore wing 2.00–2.20 × as long as its maximum width; basal cell partly setose, basal vein setose; speculum closed below; M 0.82–0.84 × as long as PM and 1.24–1.31 × as long as S, stigma large.

***Metasoma*.** Metasoma 1.54–2.00 × as long as broad, 0.76–1.05 × as long as mesosoma, 0.58–0.78 × as long as mesosoma and head. Petiole 0.86–1.11 × as long as broad. Ovipositor sheaths projecting slightly beyond apex of metasoma.

**Male.** Unknown.

##### Etymology.

The species is named in honor of Nicholai Nikolaevich Mikloukho-Maclay, a Russian explorer famous as one of the earliest scientists to settle among and study indigenous people of New Guinea. (noun in genitive case).

##### Biology.

Egg parasitoids of the hemipteran *Axiagastus* sp. (Pentatomidae).

##### Distribution.

Papua New Guinea.

##### Comments.

*Acroclisoides miklukhai* Tselikh, sp. nov. belongs to a group of species where females have a fore wing with one spot near S. This species is similar to *A. supramaculatus* Tselikh, sp. nov.; the differences between these species are given in the key.

#### ﻿ Acroclisoides nongae

Taxon classificationAnimaliaHymenopteraPteromalidae

Tselikh, Lee & Ku sp. nov.

5773C85A-3495-5494-A2A3-8DADEB057DA2

https://zoobank.org/8BCF4E80-D53F-4186-AFC3-6217094CE0CC

Figs 105–112

##### Type material.

***Holotype*** • female, Republic of Korea, “S. Korea: [GN], Sancheong-gun, Chahwang-myeon, Silmae-ri, 35.49413 N, 127.94170 E, 22.VII.2024, coll. E. Tselikh” (NIBR).

**Figures 105–112.**
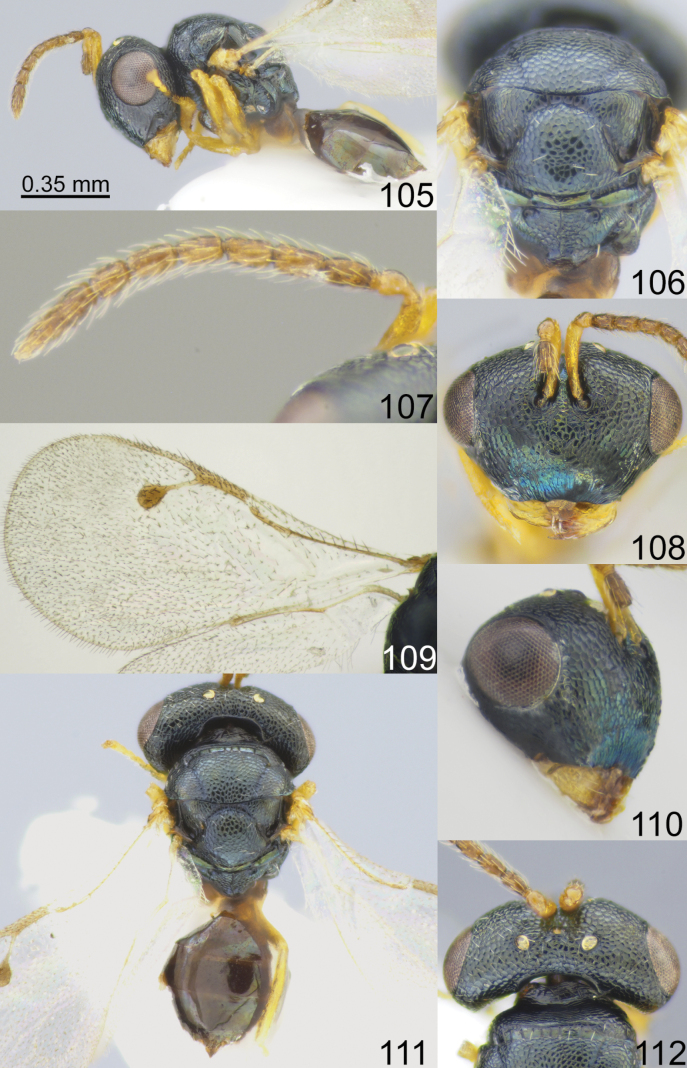
*Acroclisoides nongae* Tselikh, Lee & Ku, sp. nov., female, holotype. 105. Habitus, lateral view; 106. Mesosoma and propodeum, dorsal view; 107. Antenna; 108. Head, frontal view; 109. Fore wing; 110. Head, lateral view; 111. Habitus, dorsal view; 112. Head and pronotum, dorsal view.

##### Description.

**Female.** Body length 1.45 mm; fore wing length 1.25 mm.

***Coloration*.** Head and mesosoma dorsally black; head frontally black with diffuse metallic blue and coppery luster; antenna with scape, pedicel and anelli yellowish brown, F1–F6 and clava brown. Fore and hind coxae dark blue with diffuse metallic coppery luster, mid coxae yellowish brown, all femora and tibiae yellowish brown, tarsi yellow. Fore wing hyaline, venation brown. Metasoma dorsally dark brown with metallic violet, blue, coppery and green luster; ovipositor sheaths brown.

***Sculpture*.** Head and mesosoma reticulate; clypeus striate-reticulate; scutellum reticulate, frenal area finely reticulate; propodeum reticulate, nucha alutaceous; petiole smooth; metasoma smooth and shiny.

***Head*.** Head in dorsal view 2.21 × as broad as long and 1.36 × as broad as mesoscutum; in frontal view 1.48 × as broad as high. POL 0.95 × as long as OOL. Eye height 1.05 × eye length and 1.80 × malar space. Distance between antennal toruli and lower margin of clypeus 2.00 × distance between antennal toruli and median ocellus. Antenna with scape 0.95 × as long as eye height and as long as eye length; pedicel 1.68 × as long as broad; combined length of pedicel and flagellum 1.00 × breadth of head; F1–F6 longer than broad, F1 1.70 × as long as broad and with 2 rows of sensilla; clava 3.50 × as long as broad, with small microsetose area on C3 and C4. Lower posterior corner of gena rounded. Lower margin of clypeus concave bilaterally, in middle part weakly emarginate.

***Mesosoma*.** Mesosoma 1.29 × as long as broad. Scutellum moderately arched, 0.80 × as long as broad, frenal area differentiated by a change in sculpture. Propodeum 0.66 × as long as scutellum, without costula but with weak median carina, nucha small. Fore wing 2.01 × as long as its maximum width; basal cell partly setose, basal vein setose; speculum closed below; M 0.55 × as long as PM and 0.84 × as long as S, stigma large.

***Metasoma*.** Metasoma 1.38 × as long as broad, 0.88 × as long as mesosoma, 0.71 × as long as mesosoma and head. Petiole 0.95 × as long as broad. Ovipositor sheaths projecting slightly beyond apex of metasoma.

**Male.** Unknown.

##### Etymology.

The species is named in honor of the virtuous woman Nongae, an outstanding historical figure in Korea (noun in apposition).

##### Biology.

Unknown.

##### Distribution.

Republic of Korea.

##### Comments.

*Acroclisoides nongae* Tselikh, Lee & Ku, sp. nov. belongs to a group of species that have a hyaline fore wing. This species is similar to *A. major* Girault & Dodd; the differences between these species are given in the key.

#### ﻿ Acroclisoides quintus

Taxon classificationAnimaliaHymenopteraPteromalidae

Xiao & Huang, 2000

DDE7B666-7815-5DB3-BDA3-75351AA485B2

Figs 113–119

Acroclisoides quintus Xiao & Huang, 2000: 95–96, 98–99. Holotype female (IZAS, examined).

##### Type material.

***Holotype*** • female, China, “25.vi.1980 FUJIAN: Daoshui”, “leg. X.F. Zhao”, “*Acroclisoides quintus* Xiao & Huang, 2000 det. Xiao Hui 1998”, “Holotype”, “IOZ(E) 1221888” (IZAS).

**Figures 113–119.**
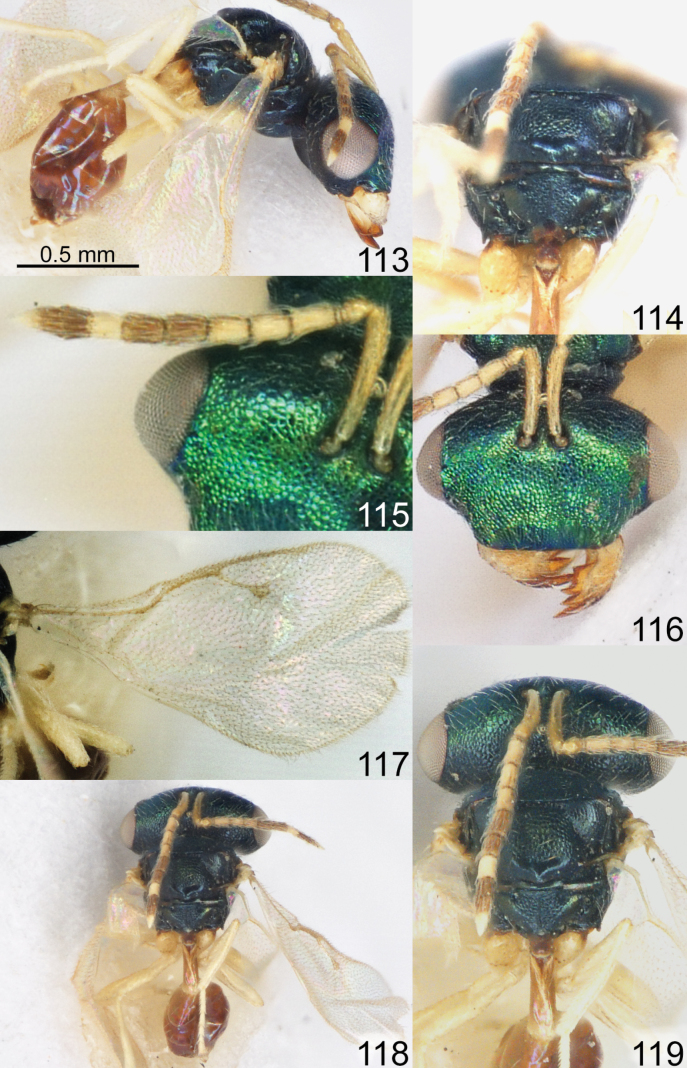
*Acroclisoides quintus* Xiao & Huang, 2000, female, holotype. 113. Habitus, lateral view; 114. Mesosoma and propodeum, dorsal view; 115. Antenna; 116. Head, frontal view; 117. Fore wing; 118. Habitus, dorsal view; 119. Head, mesosoma and propodeum, dorsal view.

##### Description.

**Female.** Body length 2.00 mm; fore wing length 1.75 mm.

***Coloration*.** Head and mesosoma dorsally dark blue with diffuse metallic green luster; head frontally green with diffuse metallic coppery luster; antenna with scape, pedicel and anelli yellowish brown, F1–F3 and F6 yellow, F4–F5 brown, clava C1–C2 brown, C3–C4 yellow. All coxae yellowish brown, all femora, tibiae and tarsi yellow. Fore wing hyaline, venation brown. Metasoma dorsally brown with metallic violet luster; ovipositor sheaths brown.

***Sculpture*.** Head, mesosoma and clypeus reticulate; scutellum reticulate, but frenal area smooth and shiny; propodeum reticulate, nucha smooth and shiny; petiole smooth; metasoma smooth and shiny.

***Head*.** Head in dorsal view 2.46 × as broad as long and 1.49 × as broad as mesoscutum; in frontal view 1.60 × as broad as high. POL 0.63 × as long as OOL. Eye height 1.11 × eye length and 2.50 × malar space. Distance between antennal toruli and lower margin of clypeus 3.25 × distance between antennal toruli and median ocellus. Antenna with scape 1.10 × as long as eye height and 1.22 × as long as eye length; pedicel 1.15 × as long as broad; combined length of pedicel and flagellum 0.93 × breadth of head; F1–F6 longer than broad, F1 1.54 × as long as broad and with 1 row of sensilla; clava 2.66 × as long as broad, with small microsetose area on C3 and C4. Lower posterior corner of gena rounded. Lower margin of clypeus not concave bilaterally, in middle part straight.

***Mesosoma*.** Mesosoma 1.16 × as long as broad. Scutellum moderately arched, 0.83 × as long as broad, frenal area differentiated by a change in sculpture. Propodeum 0.68 × as long as scutellum, without costula but with median carina, nucha not small. Fore wing 2.13 × as long as its maximum width; basal cell partly setose, basal vein setose; speculum closed below; M 0.68 × as long as PM and 1.15 × as long as S, stigma not large.

***Metasoma*.** Metasoma 2.06 × as long as broad, 1.32 × as long as mesosoma, 0.90 × as long as mesosoma and head. Petiole 0.92 × as long as broad. Ovipositor sheaths projecting slightly beyond apex of metasoma.

**Male.** Unknown.

##### Biology.

Unknown.

##### Distribution.

China (Xiao and Huang 2000).

##### Comments.

*Acroclisoides quintus* Xiao & Huang belongs to a group of species that have a hyaline fore wing, but this species is easily distinguished from the others by the yellow F1–F3 (Fig. 115) and smooth frenal area of scutellum (Figs 114, 119).

#### ﻿ Acroclisoides sativus

Taxon classificationAnimaliaHymenopteraPteromalidae

Kumar & Khan, 2012

B0257CBD-73E5-53FF-91A4-14F7110AD406

Figs 120–127

Acroclisoides sativa Kumar & Khan, 2012: 2–4. Holotype female (BUAT, not examined).

##### Material examined.

India • 8 females, 1 males, “INDIA: Srinagar ex eggs on apple 12.8.1968 M. Hayat”, “251 M”, “NHMUK 013455954”, “NHMUK 013455955”, “NHMUK 013455957”, “NHMUK 013455960” “NHMUK 013455963” (NHMUK).

**Figures 120–127.**
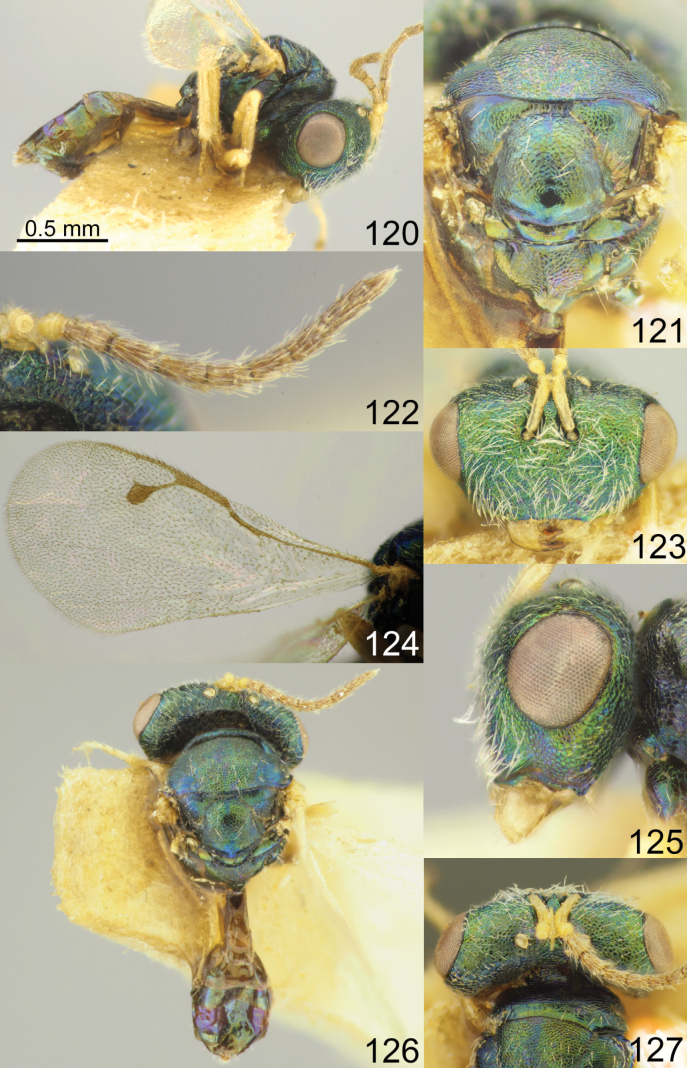
*Acroclisoides sativus* Kumar & Khan, 2012, female, not type. 120. Habitus, lateral view; 121. Mesosoma and propodeum, dorsal view; 122. Antenna; 123. Head, frontal view; 124. Fore wing; 125. Head, lateral view; 126. Habitus, dorsal view; 127. Head and pronotum, dorsal view.

##### Description.

**Female.** Body length 2.10–2.40 mm; fore wing length 2.10–2.20 mm.

***Coloration*.** Head and mesosoma metallic green blue with diffuse coppery and violet luster; antenna with scape, pedicel and anelli yellowish brown, F1–F6 and clava brown. Fore and hind coxa dark green with diffuse metallic coppery luster, mid coxae yellowish brown, all femora yellowish brown, all tibiae and tarsi yellow. Fore wing hyaline, venation brown. Metasoma dorsally dark brown with metallic violet, green and coppery luster; ovipositor sheaths brown.

***Sculpture*.** Head, clypeus and mesosoma reticulate; scutellum strongly reticulate, frenal area alutaceous and in middle part shiny, propodeum reticulate, nucha smooth and shiny; petiole smooth; metasoma smooth and shiny.

***Head*.** Head in dorsal view 2.50–2.60 × as broad as long and 1.35–1.36 × as broad as mesoscutum; in frontal view 1.72–1.73 × as broad as high. POL 0.84–0.86 × as long as OOL. Eye height 1.11–1.18 × eye length and 1.54–1.60 × malar space. Distance between antennal toruli and lower margin of clypeus 1.83–2.15 × distance between antennal toruli and median ocellus. Antenna with scape 0.80–1.11 × as long as eye height and 0.83–0.94 × as long as eye length; pedicel 1.00–1.13 × as long as broad; combined length of pedicel and flagellum 0.91 × breadth of head; F1–F6 longer than broad, F1 1.65–1.83 × as long as broad and with 2 rows of sensilla; clava 2.80–3.80 × as long as broad, with small microsetose area on C3 and C4. Lower posterior corner of gena rounded. Lower margin of clypeus concave bilaterally, in middle part weakly emarginate, near straight.

***Mesosoma*.** Mesosoma 1.19–1.22 × as long as broad. Scutellum moderately arched, 0.95–1.05 × as long as broad, frenal area differentiated by a change in sculpture. Propodeum 0.67–0.68 × as long as scutellum, without costula but with median carina, nucha not small. Fore wing 2.05–2.06 × as long as its maximum width; basal cell and basal vein setose; speculum closed below; M 0.76–0.82 × as long as PM and 1.05–1.12 × as long as S, stigma large.

***Metasoma*.** Metasoma 2.13–2.26 × as long as broad, 1.08–1.20 × as long as mesosoma, 0.87–0.88 × as long as mesosoma and head. Petiole 0.83–1.00 × as long as broad. Ovipositor sheaths projecting slightly beyond apex of metasoma.

**Male.** Body length 2.00 mm; fore wing length 1.95 mm. Fore wing with M 1.20 × as long as S. Metasoma 2.77 × as long as broad, 1.33 × as long as mesosoma, 1.10 × as long as mesosoma and head. Otherwise, similar to female.

##### Biology.

Unknown.

##### Distribution.

India (Kumar and Khan 2012).

##### Comments.

*Acroclisoides sativus* Kumar & Khan belongs to a group of species that have a hyaline fore wing in both sexes. This species is similar to *A. luzonensis* Gahan, *A. major* Girault & Dodd and *A. nongae* Tselikh, Lee & Ku, sp. nov.; the differences between these species are given in the key.

#### ﻿ Acroclisoides simbis

Taxon classificationAnimaliaHymenopteraPteromalidae

Tselikh & Mitroiu sp. nov.

1A121FBF-E7D4-57D4-8C98-041F23FEBEC8

https://zoobank.org/FA304056-F1D3-4943-8133-1F2AED082546

Figs 128–135

##### Type material.

***Holotype*** • female, Democratic Republic of the Congo, “REP. CONGO: Dpt. Pool Iboubikro, Lesio-Louna Pk 3°16'11"N, 15°28'10"E 29.vii.2008 M Sharkey MT”, “Univ. Calif. Riverside Ent. Res. Museum UCRC ENT 280631”, “*Acroclisoides* Det. R.A. Burks” (UCR). ***Paratypes*** • 2 females, Malawi, “Malawi: Kasungu Mtunthama, vii–ix. 1983, J. Feehan” (NHMUK).

**Figures 128–135.**
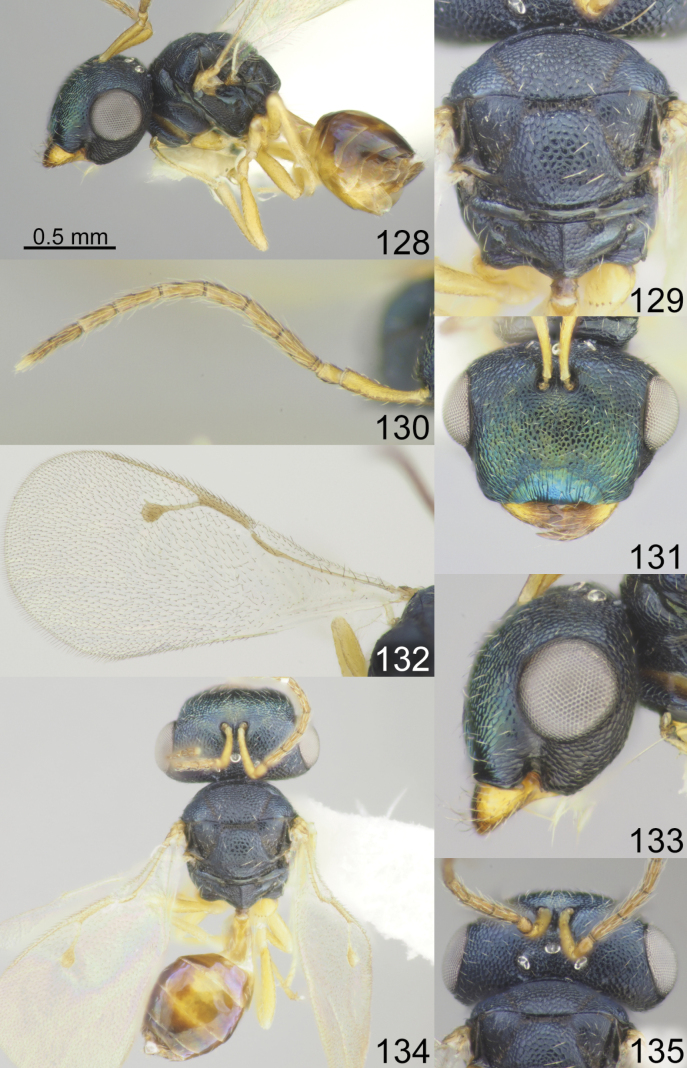
*Acroclisoides simbis* Tselikh & Mitroiu, sp. nov., female, holotype. 128. Habitus, lateral view; 129. Mesosoma and propodeum, dorsal view; 130. Antenna; 131. Head, frontal view; 132. Fore wing; 133. Head, lateral view; 134. Habitus, dorsal view; 135. Head and pronotum, dorsal view.

##### Description.

**Female.** Body length 1.90 mm; fore wing length 1.70 mm.

***Coloration*.** Head and mesosoma dorsally dark blue; head frontally blue green with diffuse metallic coppery luster; antenna with scape and pedicel yellowish brown, anelli and F1–F6 brown, clava with C1–C2 brown and C3–C4 yellow or whitish. All coxae and femora yellow or yellowish brown, all tibiae and tarsi yellow. Fore wing hyaline, venation brown. Metasoma dorsally brown or yellowish brown in the middle, with metallic violet and blue luster; ovipositor sheaths brown.

***Sculpture*.** Head and mesosoma reticulate; clypeus striate-reticulate; scutellum strongly reticulate, frenal area finely reticulate; propodeum reticulate, nucha alutaceous; petiole smooth; metasoma smooth and shiny.

***Head*.** Head in dorsal view 2.00–2.37 × as broad as long and 1.34–1.47 × as broad as mesoscutum; in frontal view 1.47–1.55 × as broad as high. POL 0.66–0.72 × as long as OOL. Eye height 1.04 × eye length and 2.00 × malar space. Distance between antennal toruli and lower margin of clypeus 3.83–4.10 × distance between antennal toruli and median ocellus. Antenna with scape 1.06–1.14 × as long as eye height and 1.11–1.24 × as long as eye length; pedicel 1.33 × as long as broad; combined length of pedicel and flagellum 1.12–1.20 × breadth of head; F1–F6 longer than broad, F1 2.22–2.54 × as long as broad and with 2–3 rows of sensilla; clava 4.10–4.30× as long as broad, with small microsetose area on C3 and C4. Lower posterior corner of gena rounded. Lower margin of clypeus concave bilaterally, in middle part weakly rounded.

***Mesosoma*.** Mesosoma 1.08–1.17 × as long as broad. Scutellum moderately arched, 0.84 × as long as broad, frenal area differentiated by a change in sculpture. Propodeum 0.66–0.71 × as long as scutellum, without costula but with median carina, nucha small. Fore wing 2.08–2.09 × as long as its maximum width; basal cell with 7–9 setae, basal vein setose; speculum closed below; M 0.58–0.63 × as long as PM and as long as S, stigma large.

***Metasoma*.** Metasoma 1.73–1.77 × as long as broad, 1.29–1.32 × as long as mesosoma, 0.83–0.92 × as long as mesosoma and head. Petiole as long as broad. Ovipositor sheaths projecting slightly beyond apex of metasoma.

**Male.** Unknown.

##### Etymology.

The species is named after the African nature spirits – the simbis (noun in apposition).

##### Biology.

Unknown.

##### Distribution.

Democratic Republic of the Congo, Malawi.

##### Comments.

*Acroclisoides simbis* Tselikh & Mitroiu, sp. nov. belongs to a group of species that have a hyaline fore wing. This species is similar to *A. africanus* Ferrière; the differences between these species are given in the key.

#### ﻿ Acroclisoides sinicus

Taxon classificationAnimaliaHymenopteraPteromalidae

(Huang & Liao, 1988)

FCF2AFBF-AAF4-5980-B84F-C2178ABDB81E

Figs 136–143

Neocoruna sinica Huang & Liao, 1988: 427, 428. Holotype female (IZAS, not examined).
Acroclisoides sinica (Huang & Liao, 1988); combination by Xiao and Huang (2000): 95.
Acroclisoides solus Grissell & Smith, 2006: 925, 926–928. Holotype female (USNM, examined); synonymy by Sabbatini Peverieri et al. 2019: 135.

##### Type material examined.

***Paratypes*** • 1 female, China, “Beijing, Ying Tao Gou 1984.IX.13”, “*Neocoruna sinica* Huang”, “PARATYPE”, “*Acroclisoides sinica* (Huang & Liao, 1988) Comb. Xiao & Huang Jan. 1999”, “IOZ(E) 1220073” (IZAS) • 1 female, China, “Beijing, Ying Tao Gou 1984.IX.13”, “*Neocoruna sinica* Huang”, “PARATYPE”, “*Acroclisoides sinica* (Huang) det. Huang 1992”, “*Acroclisoides* Det. Bouček, 1992”, “NHMUK 013456026” (NHMUK) • 1 female, China, “Beijing, Ying Tao Gou 1983.IX.25”, “*Neocoruna sinica* Huang”, “PARATYPE”, “*Acroclisoides sinicus* (Huang) Det. Z. Bouček, 1993”, “NHMUK(E) #953696”, “NHMUK 013456025” (NHMUK). ***Allotype*** • 1 male, China, “Beijing, Ying Tao Gou 1984.IX.13”, “*Neocoruna sinica* Huang”, “ALLOTYPE”, “*Acroclisoides sinica* (Huang & Liao, 1988) Comb. Xiao & Huang Jan. 1999”, “IOZ(E) 1220071” (IZAS).

**Figures 136–143.**
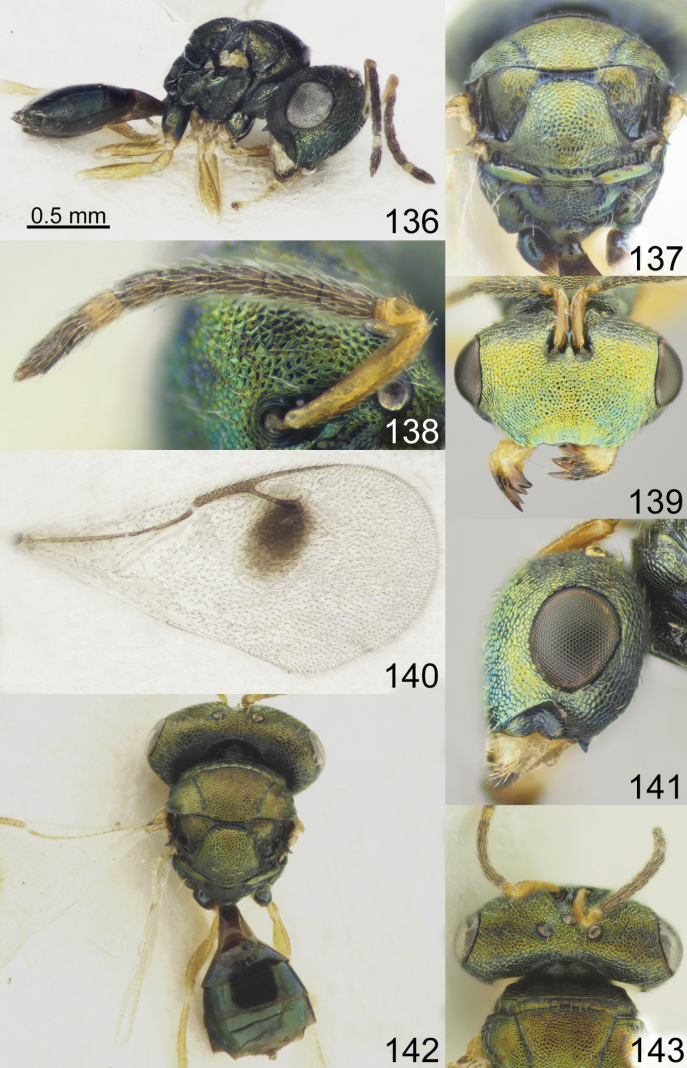
*Acroclisoides sinicus* (Huang & Liao, 1988), female, paratype. 136. Habitus, lateral view; 137. Mesosoma and propodeum, dorsal view; 138. Antenna; 139. Head, frontal view; 140. Fore wing; 141. Head, lateral view; 142. Habitus, dorsal view; 143. Head and pronotum, dorsal view.

##### Additional material examined.

Japan • 1 female, “Ibaraki Pref., Tsukuba City, forest, 10.X.1999, coll. S. Belokobylskij” (ZISP). Republic of Korea • 1 female, “S. KOREA: Gyeongsangnam-do, Sancheong-gun, 30 km NNW Jinju, forest, meadow, h=800 m 16.06.2002 S. Belokobylskij” (ZISP) • 1 female, “KOREA [GB] Gyeongju-si Hyeongok-myeon Namsa-ri (M. T.) 25.viii–2.ix.2005 J.T. Mun (SNU)” (SMNE) • 1 female, “KOREA [GW] Wonju-si Heungeop-myeon Maeji-ri 234 Yonseidae (M.T.) 31.vii–5.ix.2014 H.Y. Han” (SMNE) • 1 female, “Korea (GB) Taeha-ri Hakpo, Seo-Myeon, Ulleung-gun IX.1–IX.8 2017 (Malaise Trap) Ku Deokseo” (SMNE) • 1 female, “S. KOREA Soheul-eup, Pocheon-si, Gyeonggi-do 28.iv–10.v.2017 Kim, Kim, Nam 37°45'24.1"N, 127°09'46.1"E” (SMNE) • 2 females, “(GB), Juwangsan-myeon, Cheongsong-gun, Juwangsan SW, 12.V.2022, coll. J.H. Lee” (SMNE) • 1 female, “Korea (GN) Gaseon-ri, IIbanseong-myeon, Jinju-si VII.16.–VIII.13.2022 (malaise Trap) An Tae-Ho” (SMNE) • 2 females, “Buk-myeon, Inje-gun, (Mt) Seoraksan, 26.VIII.2022, coll. J.H. Lee” (SMNE) • 1 female, “Daegwallyeong-myeon, Pyeongchang-gun, Daegwallyeong Pass, 28.VIII.2022, coll. J.H. Lee” (SMNE) • 1 female, “(GN), Sirubong Peak, Jinhae-gu, Changwon-si, sweeping, 19.IX.2022, coll. J.H. Lee” (SMNE) • 6 females, “S. Korea: [GN], Geochang-gun, Namsang-myeon, Jeoncheok-ri, 35°37'15.3"N, 127°57'51.4"E, 21.VII.2024, coll. E. Tselikh” (ZISP) • 3 females, “S. Korea: [GN], Jinju-si, Geumsan-myeon, Galjeon-ri, 35.18906N, 128.1779E, 25.VII.2024 coll. Tselikh” (ZISP) • 2 females, “South Korea, (GN), Geochang-gun, Science Museum Natural Enemy, 31.VII.2024, coll. E. Tselikh” (ZISP) • 2 females, “South Korea: [GN], Goseong-gun, Gaecheon-myeon, Bukpyeong-ri, 35.08368N, 128.2578E, 03.VIII.2024, coll. E. Tselikh” (ZISP) • 20 females, “South Korea, (GN), Daegu, Dalseong-gun, Yang-ri, 35.71331N, 128.51140E, 5, 11.08.2024, coll.S. Belokobylskij, V. Chemyreva, E. Tselikh” (ZISP, SMNE). Russia • 1 female, “Primorskii Reg., Anisimovka, forest, 14–15.VIII.2006 coll. S. Belokobylskij” (ZISP). Vietnam • 1 female, “Vinh Phuc Pr., Me Linh District, Ngoc Thanh Tam Dao foothill, 21°24'N, 105°43'E, 12–13.V.2002 coll. S. Belokobylskij” (ZISP).

##### Description.

**Female.** Body length 1.60–2.05 mm; fore wing length 1.50–1.90 mm.

***Coloration*.** Head and mesosoma green with diffuse metallic coppery luster, propodeum dorsally dark blue green with diffuse metallic coppery luster; antenna with scape, pedicel and anelli yellowish brown, F1–F5 and clava brown, F6 yellow. Fore and hind coxae dark green with diffuse metallic coppery luster, mid coxae yellowish brown, all femora yellowish brown, all tibiae and tarsi yellow. Fore wing with one spot near S, venation brown. Metasoma dorsally dark brown with metallic violet, green and coppery luster; ovipositor sheaths brown.

***Sculpture*.** Head and mesosoma reticulate; clypeus striate-reticulate; scutellum, frenal area and propodeum reticulate, nucha finely reticulate; petiole smooth; metasoma smooth and shiny.

***Head*.** Head in dorsal view 2.27–2.30 × as broad as long and 1.39–1.42 × as broad as mesoscutum; in frontal view 1.47–1.67 × as broad as high. POL 0.56–0.57 × as long as OOL. Eye height 1.09–1.21 × eye length and 1.38–1.65 × malar space. Distance between antennal toruli and lower margin of clypeus 2.70–3.00 × distance between antennal toruli and median ocellus. Antenna with scape 0.94–1.05 × as long as eye height and 1.14–1.15 × as long as eye length; pedicel 1.11–1.35 × as long as broad; combined length of pedicel and flagellum 0.78–0.80 × breadth of head; F1–F6 longer than broad, F1 1.78–2.10 × as long as broad and with 2–3 rows of sensilla; clava 1.71–2.26 × as long as broad, with small microsetose area on C3 and C4. Lower posterior corner of gena with sharp spine. Lower margin of clypeus concave bilaterally, in middle broadly emarginate.

***Mesosoma*.** Mesosoma 1.19–1.20 × as long as broad. Scutellum moderately arched, 0.78–0.81 × as long as broad, frenal area differentiated by sculpture. Propodeum 0.59–0.70 × as long as scutellum, without costula but with median carina, nucha not small. Fore wing 1.93–2.14 × as long as its maximum width; basal cell partly setose, basal vein setose; speculum closed below; M 0.77–0.79 × as long as PM and 1.13–1.15 × as long as S, stigma small.

***Metasoma*.** Metasoma 1.50–1.60 × as long as broad, 0.93–1.00 × as long as mesosoma, 0.77–0.79 × as long as mesosoma and head. Petiole 0.90–1.00 × as long as broad. Ovipositor sheaths projecting slightly beyond apex of metasoma.

**Male.** Body length 1.20 mm; fore wing length 1.15 mm. Head posterior to malar space striate reticulate. POL 0.75 × as long as OOL. Distance between antennal toruli and lower margin of clypeus 2.20 × distance between antennal toruli and median ocellus. Fore wing with M 1.25 × as long as S. Otherwise, similar to female.

##### Biology.

Unknown.

##### Distribution.

China (Huang and Liao 1988; UCD Community 2025), Japan (new record), Republic of Korea (Lee et al. 2019), Russia (Tselikh 2016), Vietnam (new record).

##### Comments.

*Acroclisoides sinicus* (Huang & Liao) belongs to a group of species where females have a fore wing with one spot near S. It is very close to *A. solus* (see comments under that species) and can be separated from it by the characters given in the key.

#### ﻿ Acroclisoides solus

Taxon classificationAnimaliaHymenopteraPteromalidae

Grissell & Smith, 2006 stat. rev.

3C082225-73DB-5D4E-AB81-7BAE52E2BD37

Figs 144–151

Acroclisoides solus Grissell & Smith, 2006: 925, 926–928. Holotype female (USNM, examined).

##### Type material examined.

***Holotype*** • female, USA: “West Virginia, Hardy County, 3 mi. NE Mathias, 38°55'N, 78°49'E, 30.VII–12.VIII.2004 coll. D.R. Smith Malaise trap” (USNM).

**Figures 144–151.**
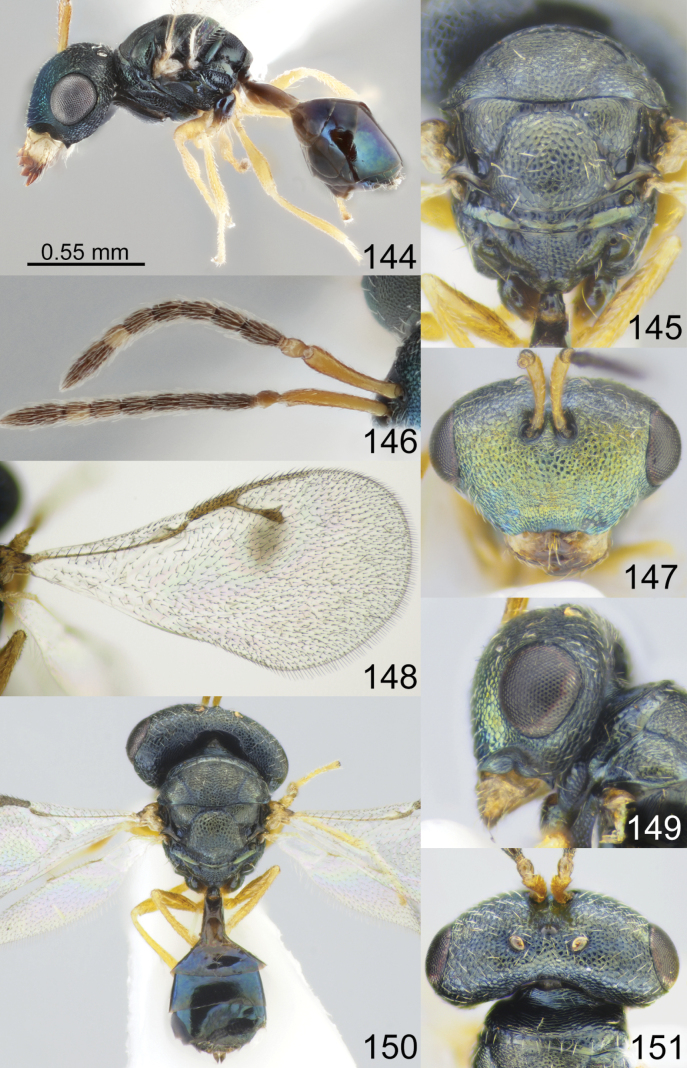
*Acroclisoides solus* Grissell & Smith, 2006, female, holotype 144, 146. and female, not type 145, 147–151. 144. Habitus, lateral view; 145. Mesosoma and propodeum, dorsal view; 146. Antenna; 147. Head, frontal view; 148. Fore wing; 149. Head, lateral view; 150. Habitus, dorsal view; 151. Head and pronotum, dorsal view.

##### Additional material examined.

China • 25 females, 1 male, “CHINA: Beijing Province Haidian, Lengquan village 40°01'08"N, 116°13'10"E 27.VI.2013, on mulberry”, “Ex: *Plautia fimbriata* eggs (primary host most likely *Trissolcus plautiae* CABI label: FCP-P591”, “FCP-P591” (CNC) • 3 females, 1 male, “CHINA: Beijing Province Haidian, Lengquan village 40°01'08"N, 116°13'10"E 4.VII.2013, on mulberry”, “Ex: *Plautia fimbriata* eggs (primary host most likely *Trissolcus plautiae* CABI label: FCP-P671”, “FCP-P671” (ZISP). Japan • 3 females, “JAPAN: Fukuoka City eggs *Plautia stali* 3.IX.1975 M. Miyahara (no. 1, Tachikawa)”, “Acroclisoides ? luzonensis Gah. det. Z. Bouček, 1976”, “NHMUK 013455966” (NHMUK) • 2 females, “Japan, Honshu, Kobe Hyo-go Pref., Rokka Mts. Maya Mt., coll. S. Belokobylskij 24.VII.2005” (ZISP). Republic of Korea • 1 female, “S. Korea [GB] Yeongyang-gun, Irwol-myeon, Mt. IIwol-san, 36°48'29"N, 129°05'25"E, 14.VII.2015 coll. E. Tselikh” (ZISP) • 1 female, “Korea (GB) Bongwha-gun, Seokpo-myeon, Seokpo-ri, Malaise trap, 31.IX.2016, coll. J.H. Lee” (SMNE) • 1 female, “Soheul-eup, Pocheon-si, Gyeonggi-do, 29.V.2015, Park, Choi, Nam, Shin, Kim, 37°45'29.2"N 127°10'0.4"E” (SMNE) • 3 females, “Soheul-eup, Pocheon-si, Gyeonggi-do, 12.VI–30.VI.2017, Kim, Kim, Nam, 37°45'1.6"N 127°08'34.9"E” (SMNE) • 1 female, “(GB), Taeha-ri Hakpo, Seo-Myeon, Ulleung-gun, VII.1–VII.15.2017, Malaise Trap, Ku Deokseo” (SMNE) • 1 female, “(JN), Jangioa-ri, Wando-eup, Wando-gun, VIII.16–VIII.29.2020, Malaise Trap, Ku Deokseo, Lee Jaehyeon” (SMNE) • 1 female, “(GB), Dahyeon-ri, Bukhu-myeon, Andong-si, V.31–VI.16.2021, Malaise Trap, Gwon Gimyeon” (SMNE) • 1 female, “(GW), Mandae-Ri, Haean-Myeon, Yanggu-Gun, Gangwon-do, VII.5–VIII.11.2021, Malaise Trap, Y.H. Park, M.H. Kim, D.H. Park, J.Y. Kim” (SMNE) • 2 females, “(GN), Muchon-ri, Namsang-gun, Geochang-gun, VII.28–VIII.15.2021, (Malaise Trap), Lee Jaehyeon, Jeong Hyojin” • 1 female, “Korea, Gyeongsangnam-do, Geochang-gun, Namsang-myeon, Jeoncheok-ri, 35°37'15.3"N, 27°57'51.4"E, 26.VI.2022, coll. E.V. Tselikh” (ZISP) • 3 females, “Republic of Korea: Gyeongsangnam-do, Geochang-gun, 35°44'54"N, 127°56'26"E, 30.06.2022, coll. E. Tselikh” (ZISP) • 1 female, “S. Korea, (GB), JuWangSan-myeon, Cheongsong-gun, (Mt), JuWangSan SW, 18.VI.2023, coll. J.H. Lee” (SMNE) • 2 females, “S. Korea: [GN], Geochang-gun, Mari-myeon, Yeongseung-ri, 35.714060N 127.876007E, 06.VII.2023, coll. E.V. Tselikh” (ZISP) • 3 females, “South Korea, (GN), Geochang-gun, Science Museum Natural Enemy, 20.VII.2024, coll. E. Tselikh” (ZISP) • 6 females, “S. Korea: [GN], Sancheong-gun, Chahwang-myeon, Silmae-ri, 35.49413N 127.94170E, 22.VII.2024, coll. V. Chemyreva” (ZISP) • 1 female, “S. Korea: [GN], Jinju-si, Geumsan-myeon, Galjeon-ri, 35.18906N, 128.1779E, 25.VII.2024 coll. Tselikh” (ZISP) • 2 females “S. Korea, GN, Goseong -gun, Hail-myeon, Suyang-ri, 34°58'34.8"N, 128°12'08.3"E, 7.VIII.2024, coll. Tselikh” (ZISP) • 12 females “South Korea, (GN), Daegu, Dalseong-gun, Yang-ri, 35.71331N, 128.51140E, 9.08.2024, coll E. Tselikh” (NHMUK, ZISP). Russia • 1 female, “RUSSIA: Amur Prov., 40 km SW Svobodny, 27–29.07.2003 coll. S. Belokobylskij” (ZISP) • 2 females, “RUSSIA: Amur Prov., Khingan Reserve, 3 km. E Uril, 3–4.08.2022 coll. O. Kosheleva” (ZISP) • 1 female, “RUSSIA: Primorskii Reg., 30 km E Spassk, forest, 7.07.1993 coll. S. Belokobylskij” (ZISP) • 2 females, “RUSSIA: Primorskii Reg., Novokachalinsk, Khanka Lake, 12–16.08.2003 coll. S. Belokobylskij” (ZISP) • 1 female, “RUSSIA: Primorskii Reg., 18 km SE Lazo, Lazovsky Reserve, forest, 24–29.08.2006 coll. S. Belokobylskij” (ZISP). USA • 1 female, “Ohio, Geauga Co. Holden Arboretum Stibbins Gulch Aug. 14, 2004 sweep – T. Pucci”, “Univ. Calif. Riverside Ent. Res. Museum UCRC ENT 508661” (UCR) • 1 female, “USA: NY: Seneca Co. 4.5 mi. SW Lodi, 200 m 42°33'45"N, 76°52'27"E 30.vii–14.viii.2010 G. Loeb. S. Triapitsyn, vinevard MT”, “Univ. Calif. Riverside Ent. Res. Museum UCRC ENT 284394” (UCR).

##### Description.

**Female.** Body length 1.70–1.80 mm; fore wing length 1.40–1.50 mm.

***Coloration*.** Head and mesosoma dorsally dark blue with diffuse metallic coppery luster, head frontally green with diffuse metallic coppery luster, antenna with scape, pedicel and anelli yellowish brown, F1–F5 and clava brown, F6 yellow. Fore and hind coxa dark blue with diffuse metallic violet luster, mid coxae yellowish brown, all femora and tibiae yellowish brown, all tarsi yellow. Fore wing with one spot near S, venation brown. Propodeum dorsally dark blue with diffuse metallic coppery luster. Metasoma dorsally dark brown with metallic blue and violet luster; ovipositor sheaths brown.

***Sculpture*.** Head and mesosoma reticulate; clypeus reticulate-striate; scutellum, frenal area and propodeum reticulate, nucha alutaceous; petiole smooth; metasoma smooth and shiny.

***Head*.** Head in dorsal view 2.21–2.30 × as broad as long and 1.34–1.38 × as broad as mesoscutum; in frontal view 1.54–1.59 × as broad as high. POL 0.67–0.71 × as long as OOL. Eye height 1.10–1.12 × eye length and 1.64–1.65 × malar space. Distance between antennal toruli and lower margin of clypeus 2.80–2.87 × distance between antennal toruli and median ocellus. Antenna with scape 0.94–1.00 × as long as eye height and 1.00–1.06 × as long as eye length; pedicel 1.20–1.25 × as long as broad; combined length of pedicel and flagellum 0.88–0.90 × breadth of head; F1–F6 longer than broad, F1 1.25–1.75 × as long as broad and with 2 rows of sensilla; clava 2.50–3.30 × as long as broad, with small microsetose area on eC3 and C4. Lower posterior corner of gena with sharp spine. Lower margin of clypeus concave bilaterally, in middle emarginate.

***Mesosoma*.** Mesosoma 1.09–1.16 × as long as broad. Scutellum moderately arched, 0.77–0.80 × as long as broad, frenal area not clear differentiated by sculpture. Propodeum 0.60–0.80 × as long as scutellum, without costula but with median carina, nucha not small. Fore wing 2.05–2.07 × as long as its maximum width; basal cell and basal vein setose; speculum closed below; M 0.64–0.70 × as long as PM and 0.85–0.92 × as long as S, stigma small.

***Metasoma*.** Metasoma 1.62–1.85 × as long as broad, 1.16–1.33 × as long as mesosoma, 0.89–0.90 × as long as mesosoma and head. Petiole 0.90–1.10 × as long as broad. Ovipositor sheaths projecting slightly beyond apex of metasoma.

**Male.** Body length 1.50–1.60 mm; fore wing length 1.25–1.30 mm. Distance between antennal toruli and lower margin of clypeus 2.57–2.60 × distance between antennal toruli and median ocellus. Antenna with pedicel 1.40–1.45 × as long as broad. Otherwise, similar to female.

##### Biology.

Eggs parasitoid of hemipterans *Plautia fimbriata* Stål, 1865 (new host record) and *P. stali* Scott, 1874 (new host record) (Pentatomidae). Hyperparasitoids of *Trissolcus plautiae* (Watanabe, 1954) (Scelionidae) (new host record).

##### Distribution.

China, Japan, Russia (new records), Italy, Republic of Korea, Switzerland (as *A. sinicus*), USA (Grissell and Smith 2006; Sabbatini Peverieri et al. 2019).

##### Comments.

*Acroclisoides solus* Grissell & Smith was synonymized with *A. sinicus* (Huang & Liao) by Sabbatini Peverieri et al. (2019), based on morphological and molecular evidence. However, after examining many additional specimens (see above), we noticed that with a few exceptions they can be grouped in two morphs based on the characters mentioned in the key. Moreover, no type specimen of *A. sinicus* was sequenced in the mentioned study, so based on these findings we prefer to treat these specimens as two different species until further evidence proves otherwise. The specimens listed in Sabbatini Peverieri et al. (2019) and deposited in MICO, not repeated here, should be treated as *A. solus*, except the following one that probably belongs to *A. sinicus*: “1♀ S. Korea: Chungbuk, Okcheon-gun, Bougimyeon, Soesan-li, 150 m, Malaise trap, 10.ix–03.x.2004, 36°16.594'N, 127°36.742'E, Tripotin rec. (MICO)”. The species was introduced both in Europe and North America from Asia, but its exact origin is still unknown. *Acroclisoides solus* belongs to a group of species where females have a fore wing with one spot near S However, two females from South Korea listed in Sabbatini Peverieri et al. (2019) had hyaline fore wings even if they were genetically very close to the spotted females. Thus they run to *A. bicolor* in the present key; nevertheless they should be regarded as exceptions and can be separated from *A. bicolor* by the shape of gena (with a distinct spine in *A. solus* and without in *A. bicolor*).

#### ﻿ Acroclisoides spilopterus

Taxon classificationAnimaliaHymenopteraPteromalidae

(Masi, 1917)

E97EC8AE-8F34-54AB-84DF-320171F31B35

Figs 152–159

Pachycreptis spilopterus Masi, 1917: 185–186. Lectotype female (NHMUK, examined).

##### Type material examined.

***Lectotype*** • female, Seychelles, “Mahe, '08-9. Seychelles Exp.”, “Percy Sladen Trust Exped. B. M. 1913-170.”, “*Pachycrepis spilopterus* ♀ Masi”, “LECTOTYPE”, “B.M. TYPE HYM. 5.858”, “NHMUK 010370216” (NHMUK). ***Paralectotype*** • 1 female, Seychelles, “Mahe, 08-9. Seychelles Exp.”, “Percy Sladen Trust Exped. B. M. 1913-170.”, “*Pachycrepis spilopterus* ♂ Masi”, “PARALECTOTYPE”, “NHMUK 013456027”, “*Acroclisoides spilopterus* (Masi) Det. Z. Bouček, 1974” (NHMUK).

**Figures 152–159.**
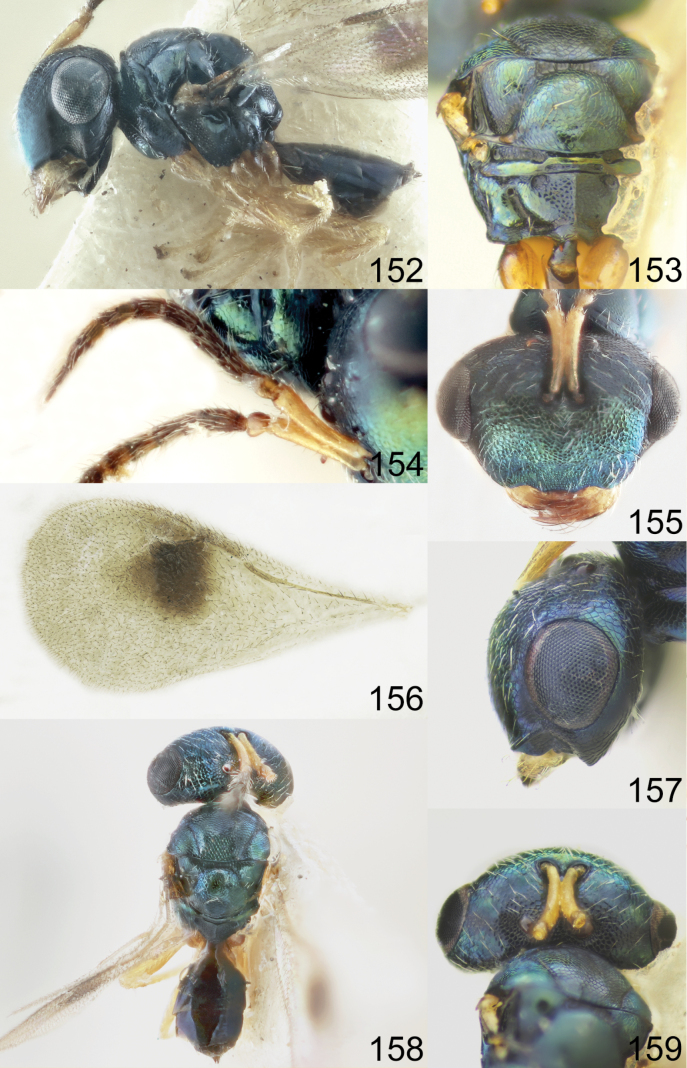
*Acroclisoides spilopterus* (Masi, 1917), female, paralectotype. 152. Habitus, lateral view; 153. Mesosoma and propodeum, dorsal view; 154. Antenna; 155. Head, frontal view; 156. Fore wing; 157. Head, lateral view; 158. Habitus, dorsal view; 159. Head and pronotum, dorsal view.

##### Description.

**Female.** Body length 1.50–1.90 mm; fore wing length 1.25–1.50 mm.

***Coloration*.** Head and mesosoma dorsally dark blue with diffuse metallic violet and green luster, head frontally blue green with metallic diffuse coppery luster, antenna with scape and pedicel yellowish brown, anelli, F1–F6 and clava brown. All coxa yellowish brown, all femora, tibiae and tarsi yellow. Fore wing with one spot near S, venation brown. Propodeum dorsally dark blue with diffuse metallic coppery and green luster. Metasoma dorsally dark brown with metallic blue and violet luster; ovipositor sheaths brown.

***Sculpture*.** Head, mesosoma and clypeus reticulate; scutellum and frenal area finely reticulate, propodeum reticulate, nucha alutaceous; petiole smooth; metasoma smooth and shiny.

***Head*.** Head in dorsal view 1.90–2.20 × as broad as long and 1.46–1.55 × as broad as mesoscutum; in frontal view 1.46–1.57 × as broad as high. POL 0.61–0.72 × as long as OOL. Eye height 1.07–1.13 × eye length and 2.10–2.28 × malar space. Distance between antennal toruli and lower margin of clypeus 2.44–2.57 × distance between antennal toruli and median ocellus. Antenna with scape 1.00–1.10 × as long as eye height and 1.00–1.06 × as long as eye length; pedicel 1.45–1.57 × as long as broad; combined length of pedicel and flagellum 1.11–1.15 × breadth of head; F1–F6 longer than broad, F1 2.00–2.25 × as long as broad and with 2 rows of sensilla; clava 2.50–2.60 × as long as broad, with small microsetose area on C3 and C4. Lower posterior corner of gena rounded. Lower margin of clypeus concave bilaterally, in middle broadly emarginate.

***Mesosoma*.** Mesosoma 1.37–1.45 × as long as broad. Scutellum moderately arched, 0.78–0.80 × as long as broad, frenal area not clearly differentiated by a change in sculpture. Propodeum 0.66–0.86 × as long as scutellum, without costula but with median carina, nucha not small. Fore wing 2.00–2.25 × as long as its maximum width; basal cell and basal vein setose; speculum closed below; M 0.57–0.70 × as long as PM and 0.90–1.10 × as long as S, stigma large.

***Metasoma*.** Metasoma 1.78–1.92 × as long as broad, 1.15–1.27 × as long as mesosoma, 0.80–0.93 × as long as mesosoma and head. Petiole 0.67–0.70 × as long as broad. Ovipositor sheaths projecting slightly beyond apex of metasoma.

**Male.** Unknown. Previously discovered males have been re-identified as females.

##### Biology.

Unknown.

##### Distribution.

Seychelles (Masi 1917).

##### Comments.

*Acroclisoides spilopterus* (Masi) belongs to a group of species where females have a fore wing with one spot near S. This species is similar to *A. supramaculatus* Tselikh, sp. nov. and *A. miklukhai* Tselikh, sp. nov.; the differences between these species are given in the key.

#### ﻿ Acroclisoides supramaculatus

Taxon classificationAnimaliaHymenopteraPteromalidae

Tselikh sp. nov.

43BBB004-378C-54E5-930A-170DBEB4B47F

https://zoobank.org/42D03ED4-2852-48AB-B785-90EED9DCC336

Figs 160–167

##### Type material.

***Holotype*** • female, Australia, “AUS., QUEENSLAND: Daintree River 23.II.84. L. Masner”, “NHMUK 013455912” (CNC). ***Paratype*** • 1 female, Australia, same data as holotype (NHMUK).

**Figures 160–167.**
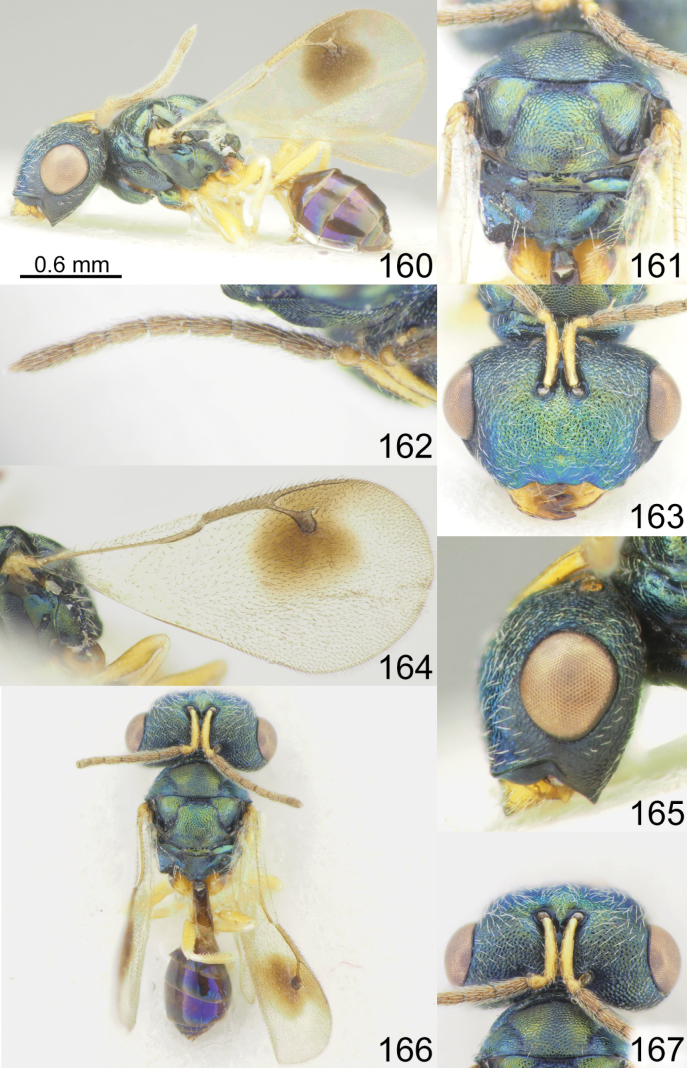
*Acroclisoides supramaculatus* Tselikh, sp. nov., female, holotype. 160. Habitus, lateral view; 161. Mesosoma and propodeum, dorsal view; 162. Antenna; 163. Head, frontal view; 164. Fore wing; 165. Head, lateral view; 166. Habitus, dorsal view; 167. Head and pronotum, dorsal view.

##### Description.

**Female.** Body length 1.75–2.40 mm; fore wing length 1.40–1.90 mm.

***Coloration*.** Head and mesosoma blue green with diffuse metallic coppery luster; antenna with scape yellow, pedicel yellowish brown, anelli, F1–F6 brown and clava brown. Fore coxae green with diffuse metallic coppery luster, mid coxae yellow, hind coxae basally brown, apically yellow, all femora, tibiae and tarsi yellow. Fore wing with one spot near S, venation brown. Metasoma dorsally brown with metallic violet and blue luster; ovipositor sheaths brown.

***Sculpture*.** Head and mesosoma reticulate; clypeus striate-reticulate; scutellum, frenal area and propodeum reticulate, nucha finely reticulate; petiole smooth; metasoma smooth and shiny.

***Head*.** Head in dorsal view 2.00–2.25 × as broad as long and 1.44–1.46 × as broad as mesoscutum; in frontal view 1.58–1.75 × as broad as high. POL 0.59–0.61 × as long as OOL. Eye height 1.05–1.08 × eye length and 1.75–1.80 × malar space. Distance between antennal toruli and lower margin of clypeus 2.27–2.30 × distance between antennal toruli and median ocellus. Antenna with scape 0.88–1.00 × as long as eye height and 1.05–1.08 × as long as eye length; pedicel 1.09–1.25 × as long as broad; combined length of pedicel and flagellum 0.88–0.95 × breadth of head; F1–F6 longer than broad, F1 2.25–2.36 × as long as broad and with 3 rows of sensilla; clava 2.80–3.09 × as long as broad, with small microsetose area on C3 and C4. Lower posterior corner of gena forming an acute angle. Lower margin of clypeus concave bilaterally, in middle part emarginate.

***Mesosoma*.** Mesosoma 1.18–1.24 × as long as broad. Scutellum moderately arched, 0.77–0.92 × as long as broad, frenal area differentiated by a change in sculpture. Propodeum 0.72–0.74 × as long as scutellum, without costula but with median carina, nucha not small. Fore wing 2.05–2.19 × as long as its maximum width; basal cell with 7–11 setae, basal vein setose; speculum partly closed below; M 0.75–0.93 × as long as PM and 1.39–1.47 × as long as S, stigma large.

***Metasoma*.** Metasoma 2.03–2.30 × as long as broad, 1.22–1.48 × as long as mesosoma, 0.83–1.13 × as long as mesosoma and head. Petiole 1.00–1.22 × as long as broad. Ovipositor sheaths projecting slightly beyond apex of metasoma.

**Male.** Unknown.

##### Etymology.

From the Latin *supra* and *macula*, referring to the large spot near S of fore wing of this species (adjective).

##### Biology.

Unknown.

##### Distribution.

Australia.

##### Comments.

*Acroclisoides supramaculatus* Tselikh, sp. nov. belongs to a group of species where females have a fore wing with one spot near S. This species is similar to *A. miklukhai* Tselikh, sp. nov.; the differences between these species are given in the key.

#### ﻿ Acroclisoides suryai

Taxon classificationAnimaliaHymenopteraPteromalidae

Tselikh sp. nov.

3CCB07F2-1DA5-5DC4-8172-980A84426555

https://zoobank.org/B936F3D4-5FEF-4227-8803-1C8FBDBFF6BE

Figs 168–175

##### Type material.

***Holotype*** • female, India, “INDIA: T. Nadu 3 km. E. Manjaler Dam”, “15–18.X.1979 J.S. Noyes B.M.”, “NHMUK 013455909” (NHMUK).

**Figures 168–175.**
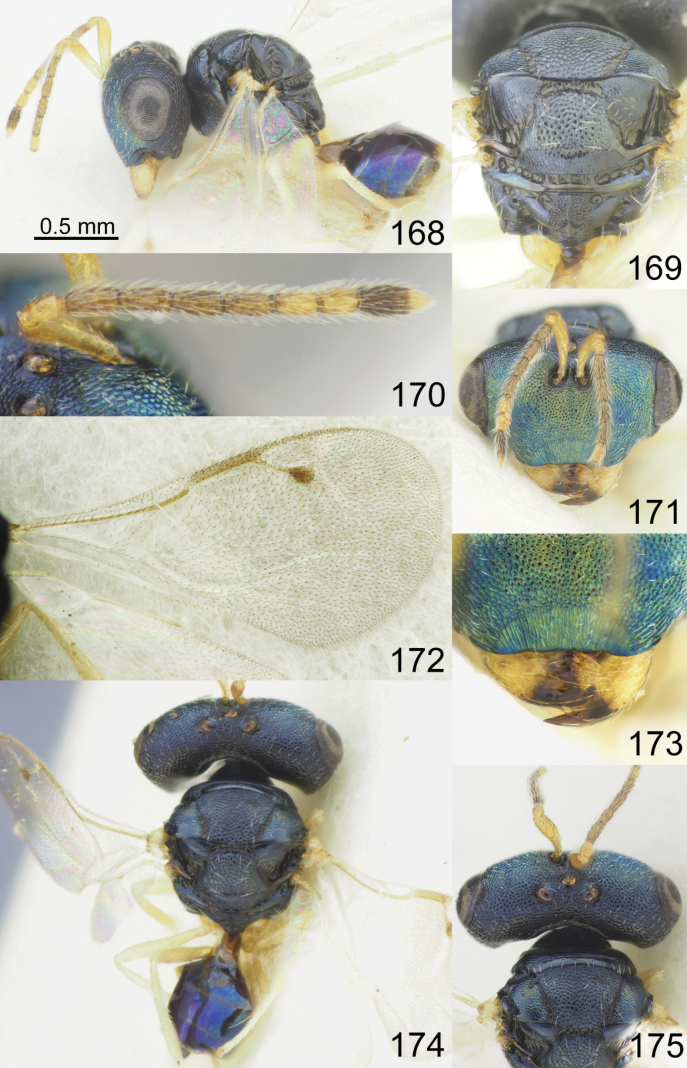
*Acroclisoides suryai* Tselikh, sp. nov., female, holotype. 168. Habitus, lateral view; 169. Mesosoma and propodeum, dorsal view; 170. Antenna; 171. Head, frontal view; 172. Fore wing; 173. Clypeus; 174. Habitus, dorsal view; 175. Head and pronotum, dorsal view.

##### Description.

**Female.** Body length 2.00 mm; fore wing length 1.90 mm.

***Coloration*.** Head and mesosoma dorsally dark blue, head frontally blue green with diffuse metallic coppery luster; antenna with scape and pedicel yellowish brown, anelli, F1–F4 brown, F5–F6 yellow, clava with C1–C2 brown, C3–C4 yellow. All coxa, femora, tibiae and tarsi yellow. Fore wing hyaline, venation brown. Metasoma dorsally brown with metallic violet and blue luster; ovipositor sheaths brown.

***Sculpture*.** Head and mesosoma reticulate; clypeus striate; scutellum strongly reticulate, frenal area finely reticulate, propodeum reticulate, nucha finely reticulate; petiole smooth; metasoma smooth and shiny.

***Head*.** Head in dorsal view 2.37 × as broad as long and 1.50 × as broad as mesoscutum; in frontal view 1.68 × as broad as high. POL 0.55 × as long as OOL. Eye height 1.22 × eye length and 1.83 × malar space. Distance between antennal toruli and lower margin of clypeus 3.46 × distance between antennal toruli and median ocellus. Antenna with scape 0.90 × as long as eye height and 1.11 × as long as eye length; pedicel 1.50 × as long as broad; combined length of pedicel and flagellum 0.68 × breadth of head; F1–F6 longer than broad, F1 2.20 × as long as broad and with 3–4 rows of sensilla; clava 3.17 × as long as broad, with small microsetose area on C3 and C4. Lower posterior corner of gena rounded. Lower margin of clypeus weakly concave bilaterally, in middle straight.

***Mesosoma*.** Mesosoma 1.22 × as long as broad. Scutellum moderately arched, 0.73 × as long as broad, frenal area differentiated by a change in sculpture. Propodeum 0.65 × as long as scutellum, without costula but with median carina, nucha small. Fore wing 2.03 × as long as its maximum width; basal cell partly setose, basal vein setose; speculum closed below; M 0.78 × as long as PM and 1.14 × as long as S, stigma large.

***Metasoma*.** Metasoma 1.18 × as long as broad, 0.82 × as long as mesosoma, 0.59 × as long as mesosoma and head. Petiole 0.83 × as long as broad. Ovipositor sheaths projecting slightly beyond apex of metasoma.

**Male.** Unknown.

##### Etymology.

The species is named in honour of Surya, the solar deity in Hinduism (noun in genitive case).

##### Biology.

Unknown.

##### Distribution.

India.

##### Comments.

*Acroclisoides suryai* Tselikh, sp. nov. belongs to a group of species that have a hyaline fore wing. This species is similar to *A. laticeps* Girault & Dodd; the differences between these species are given in the key.

#### ﻿ Acroclisoides tectacorisi

Taxon classificationAnimaliaHymenopteraPteromalidae

(Girault, 1924)

01013948-D577-58DA-AFC8-272C5B71623C

Figs 176–183

Pachycrepis tectacorisi Girault, 1924: 4. Syntype female (QMBA, examined).

##### Type material.

***Syntype*** • female, Australia, “*Acroclisoides tectacorisi* Dodd ♀”, “TYPE”, “Photographed specimen”, “TYPE Hy. 10007 A.A. Girault” (QMBA).

**Figures 176–183.**
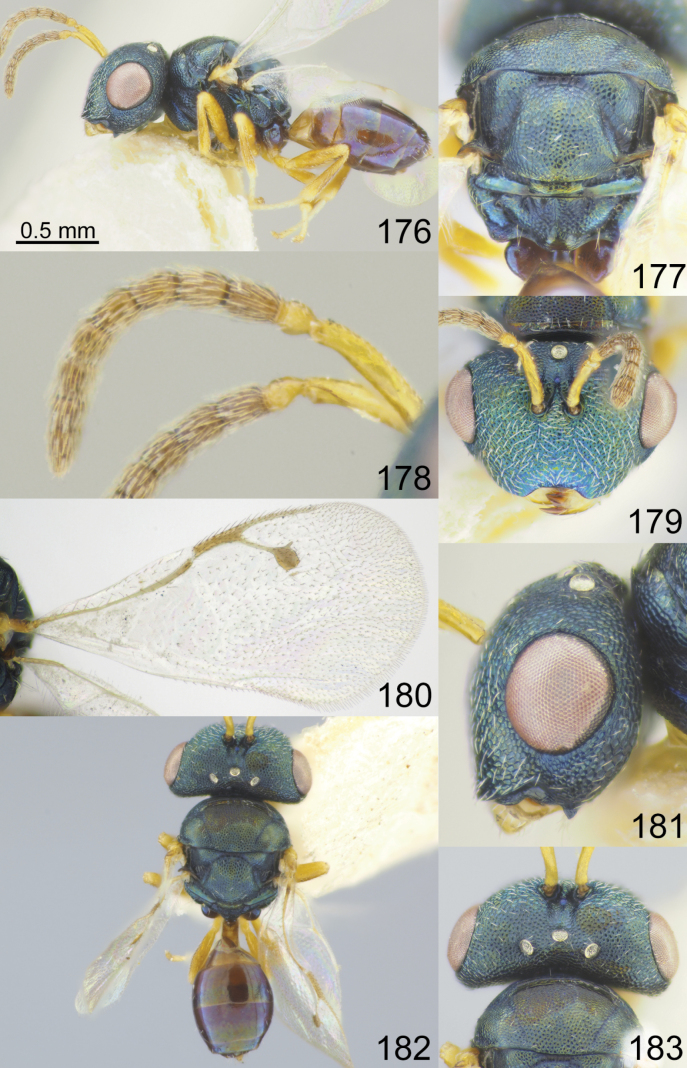
*Acroclisoides tectacorisi* (Girault, 1924), female, not type. 176. Habitus, lateral view; 177. Mesosoma and propodeum, dorsal view; 178. Antenna; 179. Head, frontal view; 180. Fore wing; 181. Head, lateral view; 182. Habitus, dorsal view; 183. Head and pronotum, dorsal view.

##### Additional material examined.

Australia • 8 females, 2 males, “S. Australia: Melton. Em. 17.xii.1924. E. Ballard.”, “Parasitic on eggs of *Tectacoris* sp.”, “Pres. By Imp. Bur. Ent. Brit. Mus. 1926–233.”, “*Pachycrepis tectacorisii*, Girault”, “NHMUK 013455922”, “NHMUK 013455946”, “NHMUK 013456028”, “NHMUK 013456029”, “NHMUK 013456030”, “NHMUK 013456032”, “NHMUK 013456033”, “NHMUK 013456034”, “NHMUK 013456035”, “NHMUK 013456036” (NHMUK, ZISP) • 2 females, 1 male, “AUSTRALIA Gunnedah NSW. 17.IV.80 N°39”, “S. Sutherland ex. Eggs of *Oechalia* sp. C.I.E. A 14389”, “*Acroclisoides tectacorisi* (Girlt) det. Z. Bouček, 1982”, “NHMUK 013455970”, “NHMUK 013455971”, “NHMUK 013455972” (NHMUK) • 1 female, “AUSTRALIA: N.S.W.: Narrabri. 24.I.1961.”, “M. Nikitin B.M. 1961–402.”, “*Pachycrepis tectacorisi*, G. 24 det. Z. Bouček, 1976”, “*Acroclisoides*”, “NHMUK 013455925” (NHMUK) • 1 female, “N.W. WALES: Mundubbera 27.IV.73 P.S.”, “ex eggs of *Biprorulus bibax* 137056”, “NHMUK 013455918” (NHMUK).

##### Description.

**Female.** Body length 1.80–2.10 mm; fore wing length 1.60–1.80 mm.

***Coloration*.** Head and mesosoma metallic blue with diffuse coppery and green luster; antenna with scape, pedicel and anelli yellowish brown, F1–F6 and clava brown. Fore and hind coxa dark blue with diffuse metallic violet luster, mid coxae yellowish brown, all femora and tibiae yellowish brown, all tarsi yellow. Fore wing hyaline, venation brown. Metasoma dorsally dark brown with metallic violet and coppery luster; ovipositor sheaths brown.

***Sculpture*.** Head and mesosoma reticulate, clypeus striate; scutellum, frenal and propodeum reticulate, nucha alutaceous; petiole smooth; metasoma smooth and shiny.

***Head*.** Head in dorsal view 2.24–2.37 × as broad as long and 1.35–1.36 × as broad as mesoscutum; in frontal view 1.46–1.56 × as broad as high. POL 0.88–0.92 × as long as OOL. Eye height 1.06–1.10 × eye length and 1.50–1.55 × malar space. Distance between antennal toruli and lower margin of clypeus 1.58–1.80 × distance between antennal toruli and median ocellus. Antenna with scape 0.82–0.88 × as long as eye height and 0.88–0.94 × as long as eye length; pedicel 1.20–1.25 × as long as broad; combined length of pedicel and flagellum 0.73–0.75 × breadth of head; F1–F6 longer than broad, F1 1.35–1.50 × as long as broad and with 2 rows of sensilla; clava 1.88–2.25 × as long as broad, with small microsetose area on C3 and C4. Lower posterior corner of gena with sharp spine. Lower margin of clypeus deeply concave bilaterally, in middle straight.

***Mesosoma*.** Mesosoma 1.23–1.26 × as long as broad. Scutellum moderately arched, 0.83–0,87 × as long as broad, frenal area differentiated by a change in sculpture. Propodeum 0.63–0.65 × as long as scutellum, without costula and with median carina, nucha small. Fore wing 1,96–2.12 × as long as its maximum width; basal cell with 0–4 setae, basal vein with 0–7 setae; speculum partly closed below; M 0.62–0.70 × as long as PM and 0.75–0.77 × as long as S, stigma large.

***Metasoma*.** Metasoma 1.61–1.62 × as long as broad, 0.87–0.98 × as long as mesosoma, 0.68–0.72 × as long as mesosoma and head. Petiole 1.00–1.10 × as long as broad. Ovipositor sheaths projecting slightly beyond apex of metasoma.

**Male.** Body length 1.45–1.60 mm; fore wing length 1.35–1.50 mm. Antenna with scape, pedicel, anelli and F1–F6 yellow; clava with C1–C2 brown, C3–C4 yellow. Metasoma 1.90–1.95 × as long as broad. Otherwise, similar to female.

##### Biology.

Egg parasitoids of hemipterans *Biprorulus bibax* Breddin, 1900, *Oechalia consocialis* Stål, 1870 and *Tectocoris diophthalmus* (Thunberg, 1783) (Pentatomidae) (Community UCD 2025).

##### Distribution.

Australia (Girault 1924).

##### Comments.

*Acroclisoides tectacorisi* (Girault) belongs to a group of species that have a hyaline fore wing, this species is easily distinguished from the others the deeply bilaterally concave lower margin of clypeus (Fig. 179).

## Supplementary Material

XML Treatment for Acroclisoides

XML Treatment for Acroclisoides africanus

XML Treatment for Acroclisoides bicolor

XML Treatment for Acroclisoides bimaculatus

XML Treatment for Acroclisoides emeljanovi

XML Treatment for Acroclisoides fusus

XML Treatment for Acroclisoides indicus

XML Treatment for Acroclisoides laticeps

XML Treatment for Acroclisoides luzonensis

XML Treatment for Acroclisoides maculatus

XML Treatment for Acroclisoides major

XML Treatment for Acroclisoides marimbae

XML Treatment for Acroclisoides megacephalus

XML Treatment for Acroclisoides miklukhai

XML Treatment for Acroclisoides nongae

XML Treatment for Acroclisoides quintus

XML Treatment for Acroclisoides sativus

XML Treatment for Acroclisoides simbis

XML Treatment for Acroclisoides sinicus

XML Treatment for Acroclisoides solus

XML Treatment for Acroclisoides spilopterus

XML Treatment for Acroclisoides supramaculatus

XML Treatment for Acroclisoides suryai

XML Treatment for Acroclisoides tectacorisi

## ﻿Additional information

### Use of AI

No use of AI was reported.

### Author ORCIDs

Ekaterina V. Tselikh https://orcid.org/0000-0002-9184-043X

Mircea-Dan Mitroiu https://orcid.org/0000-0003-1368-7721

Jaehyeon Lee https://orcid.org/0000-0002-3666-6029

Natalie Dale-Skey https://orcid.org/0000-0001-7582-0386

Deok-Seo Ku https://orcid.org/0000-0002-6274-6479

### Data availability

All of the data that support the findings of this study are available in the main text.
